# Recent Progress in the Design, Characterisation and Application of LaAlO_3_- and LaGaO_3_-Based Solid Oxide Fuel Cell Electrolytes

**DOI:** 10.3390/nano12121991

**Published:** 2022-06-09

**Authors:** Elena Filonova, Dmitry Medvedev

**Affiliations:** 1 Department of Physical and Inorganic Chemistry, Institute of Natural Sciences and Mathematics, Ural Federal University, 620002 Ekaterinburg, Russia; 2 Laboratory of Electrochemical Devices Based on Solid Oxide Proton Electrolytes, Institute of High Temperature Electrochemistry, 620660 Ekaterinburg, Russia; 3 Hydrogen Energy Laboratory, Ural Federal University, 620002 Ekaterinburg, Russia

**Keywords:** SOFCs, solid oxide fuel cells, oxygen-ion electrolytes, perovskite, LaAlO_3_, LaGaO_3_, LSGM

## Abstract

Solid oxide fuel cells (SOFCs) are efficient electrochemical devices that allow for the direct conversion of fuels (their chemical energy) into electricity. Although conventional SOFCs based on YSZ electrolytes are widely used from laboratory to commercial scales, the development of alternative ion-conducting electrolytes is of great importance for improving SOFC performance at reduced operation temperatures. The review summarizes the basic information on two representative families of oxygen-conducting electrolytes: doped lanthanum aluminates (LaAlO_3_) and lanthanum gallates (LaGaO_3_). Their preparation features, chemical stability, thermal behaviour and transport properties are thoroughly analyzed in terms of their connection with the target functional parameters of related SOFCs. The data presented here will serve as a starting point for further studies of La-based perovskites, including in the fields of solid state ionics, electrochemistry and applied energy.

## 1. Introduction

The long-term goal of a large body of relevant scientific research is to find a solution to the problem of providing industrial and domestic human needs with renewable and environmentally friendly energy [1,2]. The main fields of sustainable energy concern both the search for renewable energy sources [3,4,5] and methods for the production of ecological types of energy [6,7,8,9], which differ from traditional types based on hydrocarbon fuel [10,11,12]. The tasks relating to sustainable energy also include the development of technologies for the use of non-renewable energy sources: efficient waste-processing [13,14,15], the construction of nuclear mini-reactors [16], and the creation of energy devices based on the direct conversion of various types of energy into electrical and thermal energy [17,18,19]. A well-known device for directly converting the chemical energy of fuels into electrical energy is a fuel cell [19,20,21]. If the electrolyte in the fuel cell is a ceramic material that is permeable to oxygen ions, it is referred to as a solid oxide fuel cell (SOFC) [21,22,23,24,25].

The advantages of SOFCs are the absence of noble metals in their composition and the flexibility of fuel types [24,26,27], while the disadvantages include high operating temperatures, which lead to chemical interactions between the parts of the SOFCs [28,29] and fast degradation [30,31,32]. The high temperatures required to operate SOFCs with conventional electrolytes on the basis of yttria-stabilized zirconia (YSZ) lead to the formation of metastable phases, sealing, and thermal and chemical incompatibility with electrode materials [33,34,35].

One of the ways to solve the described problem is to decrease the operating temperature of SOFCs and develop fuel cells operating at medium- [36,37,38] and low-temperature ranges [39,40]. This has resulted in investigations into new classes of electrolytes [41,42,43,44] and the development of SOFCs enhanced with nanostructured materials [45,46]. The utilization of nanotechnologies, energy production and energy storage devices is extremely prospective due to their durability, sustainability, long lifetime, and low cost [47]. Among the alternative electrolytes used in low- and intermediate-temperature SOFCs, complex oxides with an ABO_3_-type perovskite structure have attracted specific attention due to their high efficiency in energy conversion [48,49,50]. Sr, Mg-doped lanthanum gallate (LaGaO_3_), possessing a high oxide ionic conductivity, which was established originally by Ishihara et al. in 1994 [51], was first used in SOFCs by Feng and Goodenough in 1996 [52]. Later, much more economical materials based on doped lanthanum aluminate LaAlO_3_ were reported by Fung and Chen in 2011 [53].

It is worth noting that previous generalizing works on lanthanum aluminate were aimed at the synthesis and characterization of LaAlO_3_ phosphors (published by Kaur et al. in 2013 [54]) and at some properties and applications of LaAlO_3_ not concerned with SOFCs (observed by Rizwan et al., in 2019) [55]. There is only one overview dedicated to Sr, Mg-doped LaGaO_3_ oxides as electrolytes for intermediate-temperature solid oxide fuel cells: this was published by Morales et al. in 2016 [56]. The present overview is dedicated to recent progress in the design, characterization and application of electrolyte materials for SOFCs based on the LaGaO_3_ and LaAlO_3_ complex oxides with a perovskite structure. Both these phases constitute a family of oxygen-conducting electrolytes, while other La-based perovskites (LaScO_3_, LaInO_3_, LaYO_3_, LaYbO_3_) exhibit protonic conductivity as well [49]. For this reason, scandates, indates, yttrates, and ytterbates are not considered within the present review.

**Figure 1:**
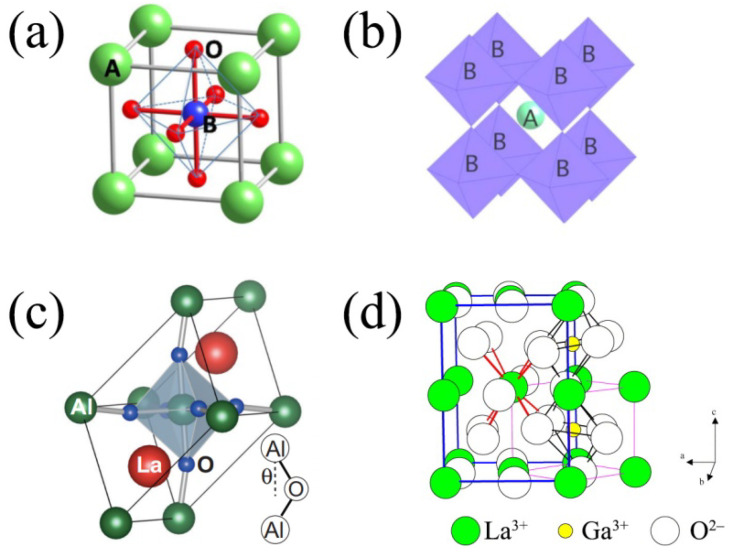
ABO_3_ perovskite structure: (**a**) B-cation centered and (**b**) A-cation centered representations; the perovskite structure of the ABO_3_ complex oxide with the B (**a**) and A (**b**) central ions [57]; (**c**) a rhombohedral crystal structure (for example, LaAlO_3_). Reproduced from [58] with permission from the American Physical Society, 2016; (**d**) an orthorhombic crystal structure (for example, LaGaO_3_) Reproduced from [59] with permission by Elsevier Ltd. (Amsterdam, The Netherlands), 2004.

A schematic image of an ABO_3_ perovskite structure is shown in Figure 1a,b. Typically, the size of A-site cations is larger than that of B-site cations, but is roughly close to that of the oxygen ions. The A-site cations are surrounded by 12 oxygen-ions in a cubo-octahedral coordination; the B-site cations are surrounded by 6 oxygen-ions in an octahedral coordination. In an ideal perovskite structure, BO_6_ octahedrons are linked at the corners, thus exhibiting the cubic *Pm3m* space group.

If the complex oxide structure differs from the ideal perovskite structure by having rhombohedral or orthorhombic distortions due to the BO_6_ octahedron arrangement, the stability of this oxide can be evaluated with the Goldsmith tolerance factor *t* equation [60] as follows:

(1) $$t=\frac{r_{A}+r_{O}}{\sqrt{2}(r_{B}+r_{O})},$$

where *r_A_*, *r_B_*, *r_O_* are the ionic radii of the A-, B-cations, and oxygen ions, respectively. If *t* is equal to 1, an ideal cubic-type perovskite structure is formed. If *t* deviates from 1, various distortions occur in the ideal perovskite structure. The first reason for such distortions is the rotation of the BO_6_ octahedron without axis deformation, which causes tilting around the large A-cations. Take, for example, the rhombohedral structure of LaAlO_3_ at room temperature presented in Figure 1c. The second reason consists of the appearance of the irregularity in the BO_6_ octahedrons due to the non-centrality of the B-site cations. Consider, for example, the orthorhombic structure of LaGaO_3_ at room temperature presented in Figure 1d.

## 2. Electrolyte Materials Based on LaAlO_3_

### 2.1. Synthesis, Structure and Morphology

For the synthesis of doped LaAlO_3_ oxides, several well-developed techniques are usually used: solid-state reaction technology [61,62,63,64], the mechanochemical route [65], co-precipitation [66,67] and organic-nitrate precursor pyrolysis [68,69,70,71,72,73,74,75].

Employing conventional solid-state reaction technology, LaAlO_3_ samples can be directly obtained from La_2_O_3_ and Al_2_O_3_. In [61], these initial reactants were ground down, homogenized in a water media, desiccated and pressed into pellets annealed at a temperature range of 780–1100 °C. Such a temperature regime allows for single-phase LaAlO_3_ samples to be prepared. A similar technology was used in work [62] to synthesize LaAl_1−*x*_Zn*_x_*O_3−δ_ (here, *δ* is the oxygen nonstoichiometry; *δ* = *x*/2 in the case of oxidation-state stable cations and one charge state difference between the host and impurity cations). As initial reagents, stoichiometric amounts of aluminium and zinc oxides were milled in ethanol. The heat treatment included five 24-h stages at a temperature range of 700–1100 °C. Single-phased LaAlO_3_ and LaAl_0.95_Zn_0.05_O_3−δ_ were obtained at 1250 and 1200 °C, respectively.

Fabian et al. [65] synthesised Ca-doped LaAlO_3_ powders using the mechanochemical method. Oxide powders of La_2_O_3_, γ-Al_2_O_3_ and CaO in appropriate proportions were milled in a planetary mill at 600 rpm. The prepared powders were pressed into disks with polyethylene glycol as a plasticizer. The LaAlO_3_ and La_1−*x*_Ca*_x_*AlO_3−δ_ pellets were sintered at 1700 and 1450 °C, respectively, to achieve a desirable ceramic densification.

LaAlO_3_ complex oxides were prepared starting from water solutions of aluminium and lanthanum chlorides with a molar ratio for the metal components of 1:1 [66]. Solutions with high and low concentrations of starting reagents were mixed with an ammonium solution serving as a precipitation agent. The obtained gels were filtered, washed with distilled water and dried twice, at 25 °C for 24 h and at 100 °C for 2 h. The prepared powders were calcined at a temperature range of 600–900 °C for 1 h. The powder obtained from the high-concentration solution was annealed at 900 °C for 2 h in air, then ground in a rotary mill with zirconia balls in dry ethanol, pressed and calcined at 1300–1500 °C for 2 h.

The most widely used technology for the preparation of LaAlO_3_ and its doped derivatives is the pyrolysis of organic-nitrate compositions, known as the sol-gel [68,69,74] or autocombustion methods (or self-propagating high-temperature synthesis, and the Pechini method) [70,71,72,73,75]. Utilizing different fuels during the pyrolysis process coupled with various annealing temperatures affects the crystallinity, powder dispersity, and ceramics density, determining the functional properties of the obtained LaAlO_3_-based ceramic materials [74,76,77].

LaAlO_3_ powders were prepared by Zhang et al. [68] from La(NO_3_)_3_·6H_2_O and Al(NO_3_)_3_·9H_2_O: they were dissolved in 2-methoxyethanol and then mixed with citric acid at a molar ratio of 1:1 to the total content of metal ions. The obtained solutions were heated and dried at 80 °C until gelatinous LaAlO_3_ precursors were obtained, which were then calcined at 600–900 °C for 2 h.

To obtain La_0.9_Sr_0.1_Al_0.97_Mg_0.03_O_3−δ_ powder, La(NO_3_)_3_·6H_2_O, Al(NO_3_)_3_·9H_2_O, Mg(NO_3_)_2_·6H_2_O, Sr(NO_3_)_2_, EDTA, C_2_H_5_NO_2_ and NH_3_·H_2_O were used in [69]. The molar ratio of glycine and EDTA to overall metal-ion content was 1.2:1:1; the ratio of NH_3_·H_2_O to EDTA was adjusted to 1.15:1. The aqueous solution of metal nitrates was prepared and heated at 80 °C, and then the EDTA-ammonia solution and glycine were added. The colourless solution was dried, and the obtained brown resin was calcined at 350 °C; it was then ground down and calcined at 600–1000 °C for 3 h. The obtained powders were finally pressed into disks followed by sintering at 1600–1700 °C for 5 h.

According to Adak and Pramanik [70], LaAlO_3_ was prepared from a 10% aqueous polyvinyl alcohol precursor that was added to a solution obtained from La_2_O_3_ (99%) dissolved in nitric acid and Al(NO_3_)_3_·9H_2_O. The organic-nitrate mixture was evaporated at 200 °C until dehydration; then, spontaneous decomposition and the formation of a voluminous black fluffy powder occurred. The obtained powders were ground down and annealed at 600–800 °C for 2 h to form a pure phase.

**Figure 2:**
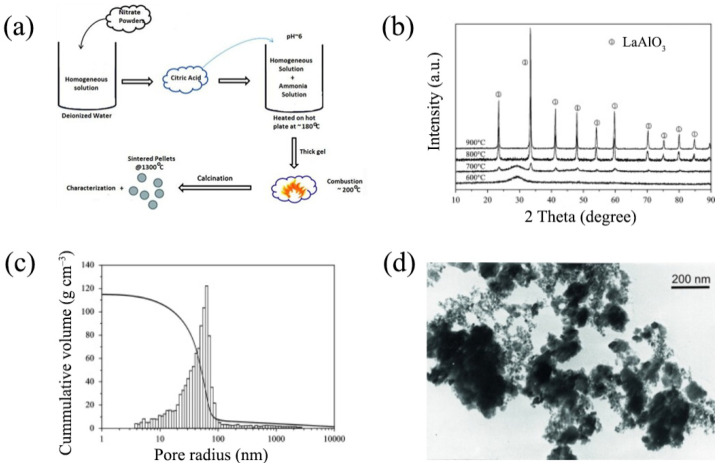
Preparation features of LaAlO_3_-based materials: (**a**) diagram of auto-combustion synthesis. Reproduced from [71] with permission from Springer Nature (Berlin/Heidelberg, Germany), 2021; (**b**) XRD patterns for LaAlO_3_ powders prepared and calcined at a temperature range of 600–900 °C for 1 h on each stage. Reproduced from [66] with permission by Elsevier Ltd., 2013; (**c**) pore size distributions of LaAlO_3_ powder bodies calcined at 900 °C for 2 h. Reproduced from [66] with permission by Elsevier Ltd., 2013; (**d**) TEM image of LaAlO_3_ powder calcined at 900 °C for 2 h. Reproduced from [66] with permission by Elsevier Ltd., 2013.

Verma et al. [71] synthesized LaAlO_3_ and La_0.9−*x*_Sr_0.1_Ba*_x_*Al_0.9_Mg_0.1_O_3−δ_ (*x* = 0.00, 0.01 and 0.03) samples from initial reagents composed of La(NO_3_)_3_·H_2_O, Sr(NO_3_)_2_, Ba(NO_3_)_2_, Al(NO_3_)_3_·6H_2_O and Mg(NO_3_)_2_·6H_2_O initial reagents. C_6_H_8_O_7_·H_2_O was used as an organic fuel. The metal nitrates and citric acid were dissolved in distilled water, resulting in the formation of a transparent solution. The pH value required for proper combustion was achieved by the addition of ammonia solution. The self-propagating synthesis method is shown in Figure 2a. The obtained powders were calcined at 700 °C for 4 h, then pressed into pellets and sintered at 1300 °C for 8 h to achieve 92-to-96% relative density, depending on the aluminate composition.

The literature shows that the annealing temperature of the precursor powders plays a significant role in complex oxide synthesis: this regulates the density of the final polycrystalline ceramic samples [78]. For practical applications, it is important to obtain LaAlO_3_-based samples with a narrow distribution of fine-grained particles. These requirements were fulfilled in [66], where a fully converted LaAlO_3_ phase was formed at relatively low temperatures. In more detail, the authors developed a co-precipitation technique enabling the formation of single-phase LaAlO_3_ powders after its calcination in air at 900 °C for 2 h (Figure 2b). A narrow particle size distribution for LaAlO_3_ powder was achieved in [66], where milling in an ethanol medium was conducted. As shown in Figure 2c, the milled LaAlO_3_ powder exhibited mono-modal pore size distribution. The TEM image (Figure 2d) demonstrates that the calcined powder consisted of isometric particles of up to 15 nm in size. The use of a precursor solution with a high concentration of metal chlorides and ammonia allowed for the researchers to realize gel homogeneity and the direct synthesis of LaAlO_3_.

A Rietveld analysis of the XRD pattern confirmed the presence of a pure perovskite phase with a rhombohedral structure, referring to the *R-3c* space group. Reference [66] calculated unit cell parameters for the LaAlO_3_ sample (*a* = 5.3556(1) Å and *c* = 13.1518(2) Å) agreed well with results from neutron powder diffraction [79]. The primitive LaAlO_3_ cell consists of two formula units, as shown in Figure 1b. The rotation of AlO_6_ octahedra is caused by changes to the *θ* angle (Al–O–Al). Above 540 °C, a phase transition from the rhombohedral to cubic structure was observed for LaAlO_3_ [79]. The cubic lattice of LaAlO_3_ with a unit cell parameter of *a* = 3.8106(1) Å corresponds to the *Pm3m* space group [79] (see Figure 1a).

Concluding the chapter about the synthesis methods of doped LaAlO_3_ oxides, from the perspective of their use in SOFCs, the co-precipitation method should be noted as the most optimal synthetic method. The co-precipitation method with a subsequent sintering of samples at 900 °C is well-approved and allows for both single-phase powders with a narrow nano-size particle distribution and ceramic samples with high relative densities to be obtained.

### 2.2. Functional Properties

**Figure 3:**
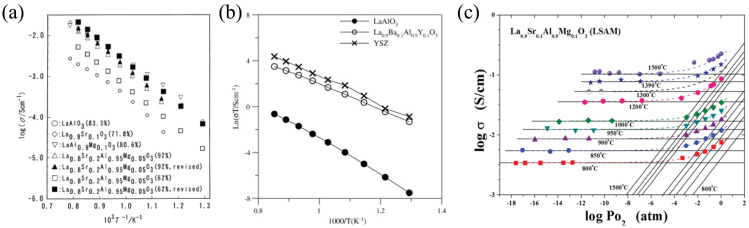
Functional properties of LaAlO_3_-doped materials: (**a**) electrical conductivity of LaAlO_3_, La_0.9_Sr_0.1_AlO_3−δ_, LaAl_0.9_Mg_0.1_O_3−δ_, La_0.8_Sr_0.2_Al_0.95_Mg_0.05_O_3−δ_ samples. Reproduced from [80] with permission by Elsevier Ltd., 2000; (**b**) electrical conductivity of LaAlO_3_, La_0.9_Ba_0.1_Al_0.9_Y_0.1_O_3−δ_, and YSZ as a reference sample. Reproduced from [53] with permission by Elsevier Ltd., 2011; (**c**) total conductivity of the La_0.9_Ba_0.1_Al_0.9_Y_0.1_O_3−δ_ ceramic as function of oxygen partial pressures [81].

LaAlO_3_, a basic (undoped) lanthanum aluminate, has very low electrical conductivity, equal to around 1 × 10^−6^ S cm^−1^ at 900 °C [75]. La-site doping of LaAlO_3_ with strontium enhances electrical conductivity because it improves the oxygen vacancy concentration responsible for oxygen-ion transport (Equation (2), [80]). Al-site modification of LaAlO_3_ with acceptor dopants (for example, magnesium) can also increase the total and ionic conductivities (see Figure 3a).

(2) $$2\mathrm{SrO}\overset{‘{\mathrm{La}}_{2}O_{3}’}{\to }2{{\mathrm{Sr}}^{\prime }}_{\mathrm{La}}+V_{O}^{••}+2O_{O}^{x}.$$

The possibility of forming good oxygen-ionic conductivity by doping LaAlO_3_ oxides has promoted studies on their potential application in SOFCs [53,65,71,82,83,84,85,86,87,88,89,90]. The co-doping strategy is a beneficial way to further increase ionic conductivity [80,82,83,87]; this is due to the fact that, along with Equation (2), an additional quantity of oxygen vacancies can be formed according to the following mechanism [80]:

(3) $$2\mathrm{MgO}\overset{‘{\mathrm{Al}}_{2}O_{3}’}{\to }2{{\mathrm{Mg}}^{\prime }}_{\mathrm{Al}}+V_{O}^{••}+2O_{O}^{x}.$$

According to the results of [53], the simultaneous doping of LaAlO_3_ with barium and yttrium drastically enhanced ionic transport. For example, the total conductivity of La_0.9_Ba_0.1_Al_0.9_Y_0.1_O_3−δ_ at 800 °C was close to that of YSZ (2 × 10^−2^ S cm^−1^), as shown in Figure 3b. There are various ways to tailor the transport properties of LaAlO_3_-based materials. For example, the doping of (La,Sr)AlO_3_ with manganese resulted in total conductivity rising due to the substitution of Mn^3+^ ions, which were transformed into Mn^2+^ and Mn^4+^ ions at the Al^3+^ position, enhancing an electronic contribution [75,84]. Therefore, co-doped (La,Sr)(Al,Mn)O_3_ is attributed to mixed ionic-electronic conductors (MIEC). The Pr-doping of (La,Sr)AlO_3_ had a positive influence on transport properties due to the suppression of grain boundary resistivity [85], and the isovalent substitution of La^3+^-ions with Sm^3+^-ions in (La,Sr)AlO_3−δ_ resulted in the formation of a pronounced mixed ion-electron conduction [88] due to the generation of more electrons than in the case of the aliovalent substitution of La^3+^ ions with Ba^2+^ ions.

**Figure A1:**
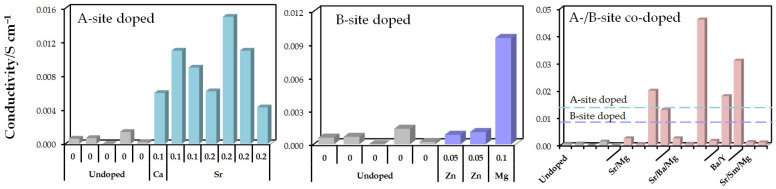
Total conductivity of the LaAlO_3_ ceramic materials at 700–900 °C depending on doping strategies. These data are taken from Table 1.

The electrical conductivity values of LaAlO_3_-based ceramic materials are summarized in Table 1. Analysis of these data confirms that the simultaneous modification of both sublattices of LaAlO_3_ results in improved conductivity compared to those reached using single doping approaches (see Figure A1). However, it should be noted that the Sr- and Mg- co-doped LaAlO_3_ materials exhibit mixed ionic-electronic conduction in air atmospheres over a wide temperature range (800–1400 °C, see Figure 3c), while predominant ionic transport occurs for more reduced atmospheres (for example, wet hydrogen). This is typical behaviour for various La-based perovskites [49] as well as for other perovskite-related ion-conducting electrolytes [91].

Thermal expansion coefficients (TECs) play an important role in material selection when seeking to avoid thermal incompatibilities between various parts of SOFCs. According to da Silva and de Miranda [75], the average TEC values for LaAlO_3_ and La_0.8_Sr_0.2_AlO_3_ were equal to around 11.4 × 10^−6^ and 9.9 × 10^−6^ K^−1^, respectively. These data confirm that the TEC values of LaAlO_3_-based materials were close to those of the conventional YSZ electrolyte, i.e., 10.9 × 10^−6^ K^−1^ [92].

The chemical compatibility of La_0.9_Sr_0.1_Al_0.97_Mg_0.03_O_3−δ_ as an electrolyte material with NiO-Ce_0.9_Gd_0.1_O_2−δ_, Sr_0.88_Y_0.08_TiO_3−δ_ and La_0.75_Sr_0.25_Cr_0.5_Mn_0.5_O_3−δ_ as anode SOFC materials was thoroughly investigated in [87] using XRD analysis and scanning electron microscopy with energy-dispersive X-ray spectroscopy. The obtained results demonstrated that Sr_0.88_Y_0.08_TiO_3−δ_ and La_0.75_Sr_0.25_Cr_0.5_Mn_0.5_O_3−δ_ interacted with La_0.9_Sr_0.1_Al_0.97_Mg_0.03_O_3−δ_ due to the interdiffusion of Sr^2+^, Ti^4+^, Mn^3+^ and Cr^3+^ cations into the La_0.9_Sr_0.1_Al_0.97_Mg_0.03_O_3−δ_ lattice. An interaction between La_0.9_Sr_0.1_Al_0.97_Mg_0.03_O_3−δ_ and NiO-Ce_0.9_Gd_0.1_O_2−δ_ at 1300 °C was not detected, which means that joint utilization is possible.

The XRD patterns of two mixtures, La_0.8_Sr_0.2_Ga_0.85_Mg_0.15_O_3−δ_/La_0.9_Sr_0.1_AlO_3−δ_ and NiO/La_0.9_Sr_0.1_AlO_3−δ_ (annealed at 1450 °C), confirmed that there were no chemical interactions between these components [93]. The authors noted that doped LaAlO_3_ materials can serve as additives to the composite electrolytes and the anode-protective layers [93]. In addition, Mn-doped LaAlO_3_ phases are considered a constituent part of the composite electrolytes, providing for the effective electrochemical oxidation of methane via ethylene and ethane [94].

### 2.3. Applications in SOFCs

**Figure 4:**
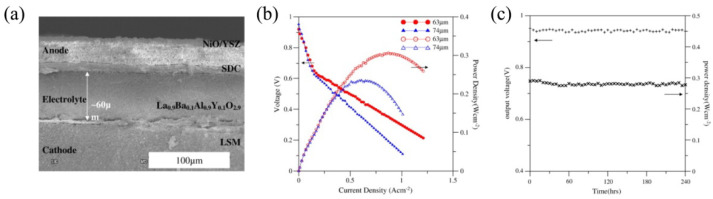
Properties of the NiO–YSZ/SDC/LBAYO/LSM SOFC: (**a**) SEM micrograph of a cell sintered at 1500 °C for 6 h; (**b**) current-voltage and current-power dependencies of a cell with an electrolyte thickness of 63 μm tested at different temperatures; (**c**) time dependencies of OCV and *P*_max_ measured at 800 °C for 10 days. These images were reproduced from [53] with permission from Elsevier Ltd., 2011.

There are fragmentary data on the application of lanthanum aluminate electrolytes in SOFCs; see Figure 4.

For example, an SOFC was fabricated with 70% NiO–30% YSZ as an anode, SDC as an interlayer, La_0.9_Ba_0.1_Al_0.9_Y_0.1_O_3−δ_ (LBAYO) as an electrolyte and LSM as a cathode, and tested in [53]. LBAYO films with thicknesses of 63 and 74 μm were electrophoretically deposited on the LSM pellets with a diameter of 25 mm and a thickness of 2 mm. The LSM substrates and the deposited LBAYO films were then annealed at 1450 °C for 2 h to achieve full electrolyte densification. The thickness of the LBAYO film varied due to increases in the applied voltage. A NiO/YSZ anode with a thickness of 40 μm was screen-printed on the LBAYO/LSM sample and then sintered at 1500 °C for 6 h. To avoid chemical interactions between the NiO and the LBAYO film, an SDC buffer layer with a thickness of 10 μm was additionally screen-printed on the LBAYO film between the electrolyte and the anode. Humidified hydrogen was used as a fuel, while air was used as an oxidant. Figure 4a presents the SEM micrograph of the NiO–YSZ/SDC/LBAYO/LSM cell, indicating that after the annealing procedure, the LBAYO film was highly densified without cracks with a uniform thickness and a strong adhesion to the LSM substrate. The open-circuit voltage (OCV) values of the fabricated cells were 0.927 and 0.953 V, while the maximum power density values were 0.306 and 0.235 W cm^−2^ for the LBAYO electrolyte layers with thicknesses of 63 and 74 μm, respectively (Figure 4b). The authors of the work attributed the sharp decrease in the cells’ voltage at a small current to the slow oxygen reduction reaction kinetics for the LSM cathode.

The long-term stability experiments demonstrated negligible degradation of the LBAYO electrolyte over 10 days. Figure 4c illustrates the time dependencies of the obtained open circuit voltage (OCV) and the maximum power density (*P*_max_) for a cell tested at 800 °C.

Another Ni-GDC/GDC/La_0.9_Sr_0.1_Al_0.97_Mg_0.03_O_3−δ_/GDC/La_0.75_Sr_0.25_FeO_3−δ_ electrolyte-supported cell was tested in [87]. For this single cell with a La_0.9_Sr_0.1_Al_0.97_Mg_0.03_O_3−δ_ electrolyte thickness of 550 μm, the OCV and *P*_max_ values at 800 °C were found to be equal to 0.925 V and 19.5 mW cm^−2^, respectively.

## 3. Electrolyte Materials Based on Doped LaGaO_3_

### 3.1. Synthesis, Structure and Morphology

**Figure 5:**
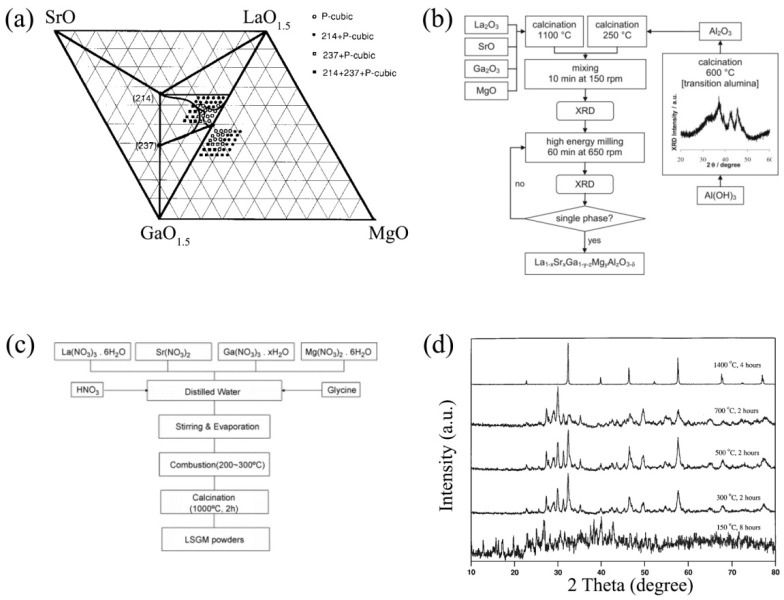
The phase and structure features of LaGaO_3_-based materials: (**a**) phase diagram of a LaO_1.5_–SrO–GaO_1.5_–MgO system up to 800 °C, P-cubic = single-phase La_1−*x*_Sr*_x_*Ga_1−*y*_Mg*_y_*O_3−δ_, 214 = LaSrGaO_4_, 237 = LaSrGa_3_O_7_. Reproduced from [95] with permission from John Wiley & Sons, Inc. (Hoboken, NJ, USA), 1998; (**b**) the scheme of mechanosynthesis for the preparation of La_1−*x*_Sr*_x_*Ga_1−*y*–*z*_Mg*_y_*Al*_z_*O_3−δ_. Reproduced from [98] with permission by Elsevier Masson SAS, 2012; (**c**) the combustion scheme synthesis for the preparation of La_1−*x*_Sr*_x_*Ga_1−*y*_Mg*_y_*O_3−δ_. Reproduced from [99] with permission by Elsevier Ltd., 2007; (**d**) XRD pattern evaluation of La_0.8_Sr_0.2_Ga_0.83_Mg_0.17_O_3−δ_ precursor powders at various calcination temperatures. Reproduced from [100] with permission from Elsevier Ltd., 1998.

Historically, La_1−*x*_Sr*_x_*Ga_1−*y*_Mg*_y_*O_3−δ_ (LSGM) oxides were the first well-studied doped materials in the LaGaO_3_ system. In 1998, Huang, Tichy and Goodenough determined the existence of single-phase La_1−*x*_Sr*_x_*Ga_1−*y*_Mg*_y_*O_3−0.5(*x*+*y*)_ perovskites while studying a LaO_1.5_-SrO-GaO_1.5_-MgO quasi-quaternary diagram [95] (see Figure 5a). This was possible due to variations in both *x* and *y* contents in a composition range of 0.05–0.30 with a step of 0.05. Sr- and Mg- co-doped LaGaO_3_ samples were prepared from La_2_O_3_, SrCO_3_, Ga_2_O_3_, and MgO using solid-state reaction technology. The obtained powders were pressed into pellets and calcined at 1250 °C for 12 h. After remilling and repressing, the final pellets were finally sintered in air at 1470 °C for 24 h and quenched in a furnace at 500 °C.

Similar conventional techniques for synthesizing La_1−*x*_Sr*_x_*Ga_1−*y*_Mg*_y_*O_3−δ_ were used in other studies [96,97]. La_0.9_Sr_0.1_Ga_0.8_Mg_0.2_O_3−δ_ samples were obtained from La_2_O_3_, SrCO_3_, Ga_2_O_3_ and MgO sources, which were mixed and sintered in a platinum crucible at 1350 °C for 12 h [96]. The annealed powder was milled with zirconia balls and dried. Then, the powder was pressed into disks and sintered at 1350 °C in air, nitrogen or oxygen atmospheres for various times ranging from 20 min to 5 h. Moure et al. [97] obtained La_0.8_Sr_0.2_Ga_0.85_Mg_0.15_O_3−δ_ and La_0.8_Sr_0.15_Ga_0.85_Mg_0.2_O_3−δ_ samples from La_2_O_3_, SrCO_3_, Ga_2_O_3_ and MgO, which were mechanochemically activated in a Pulverizette 6 Fritsch planetary mill with stainless steel balls. The mixtures were synthesized at 1300 °C for 16 h; then after milling for 2 h and sieving with a 100-μm sieve, the powders were pressed into pellets and finally sintered at 1550 °C to form the desired ceramic samples.

For the synthesis of La_0.9_Sr_0.1_Ga_1−*x*_Ni*_x_*O_3−δ_, Colomer and Kilner [101] grinded a mixture of La_2_O_3_, SrCO_3_, Ga_2_O_3_ and NiO in an agate mortar with acetone medium and then calcined them at 1000 °C for 6 h. After sieving with a 65-μm sieve, milling for 1 h, drying and secondary sieving to 65 μm, the finishing powders were pressed into disks and sintered at 1450–1500 °C for 48 h in air. The authors chose nickel as element for gallium substitution in La_0.9_Sr_0.1_GaO_3−δ_owing to the proposal about achieving a hopping conductivity among the Ni-sites.

Al-substituted La_0.95_Sr_0.05_Ga_0.9_Mg_0.1_O_3−δ_ and La_0.9_Sr_0.1_Ga_0.8_Mg_0.2_O_3−δ_ derivatives were prepared using La_2_O_3_, Ga_2_O_3_, SrO, MgO and Al_2_O_3_ [98]. Mechanosynthesis was employed in a planetary mill (Retsch PM100, PM200) with tetragonal zirconia balls, according to a scheme presented in Figure 5b. The powders were pressed into disks that were sintered at 1300–1450 °C for 2–24 h.

As can be seen, the aforementioned methods (solid-state reaction synthesis and the mechanochemical route) that were conventionally used for the preparation of La_1−*x*_Sr*_x_*Ga_1−*y*_Mg*_y_*O_3−δ_ and its derivatives have two considerable disadvantages. First, high sintering temperatures (above 1450–1500 °C) are required for full densification of the pressed pellets [51]. This can influence the production cost of the final electrolyte materials. Second, the appearance of Sr_3_La_4_O_9_, SrLaGa_3_O_7_ and/or SrLaGaO_4_ impurity phases in La_1−*x*_Sr*_x_*Ga_1−*y*_Mg*_y_*O_3−δ_ samples was frequently observed. This was due to gallium evaporation [102], which resulted in the deterioration of the gallate material’s ionic conductivity [51]. To solve the problems that arise during La_1−*x*_Sr*_x_*Ga_1−*y*_Mg*_y_*O_3−δ_ preparation, techniques based on co-precipitation [103,104], organic-nitrate precursors combustion [96,99,100,105,106,107,108,109], self-propagating, high-temperature synthesis [110,111] and spray-pyrolysis [112] were developed.

For example, La_0.8_Sr_0.2_Ga_0.8_Mg_0.2_O_3−δ_ samples were prepared with carbonate co-precipitation from La(NO_3_)_3_·6H_2_O, Sr(NO_3_)_2_, Ga(NO_3_)_3_·xH_2_O and Mg(NO_3_)_2_·6H_2_O starting reagents [103]. The resulting aqueous solution containing La^3+^, Sr^2+^, Ga^3+^ and Mg^2+^ cations was gradually dropped into an aqueous (NH_4_)_2_CO_3_ solution with heating at 70 °C. After 2 h of homogenization with continuous stirring, the formed sediments were washed, dried at 25 °C for 24 h in a N_2_ atmosphere, and finally calcined in air at 900–1300 °C for 12 h.

Huang and Goodenough [100] have reported the use of wet synthesis techniques (the sol-gel technique and the Pechini method) for forming single-phase La_0.8_Sr_0.2_Ga_0.83_Mg_0.17_O_3−δ_ materials. Solutions of La(CH_3_COO)_3_, Sr(CH_3_COO)_2_ and Mg(CH_3_COO)_2_ acetates and La(NO_3_)_3_, Sr(NO_3_)_2_, Ga(NO_3_)_3_ and Mg(NO_3_)_2_ nitrates were used in these preparation methods. During synthesis with sol-gel technology, the required amounts of metal acetates and gallium nitrate solutions were mixed by stirring. An ammonia solution was then added, forming a white gel. This was aged at 25 °C for 72 h and heated at 150 °C for 8 h upon full water evaporation. The resulting product was fired at 300, 500 and 700 °C at varying times. Using the Pechini method, La_0.8_Sr_0.2_Ga_0.83_Mg_0.17_O_3−δ_ samples were prepared from a mixture of the necessary amounts of metal nitrate solutions at 25 °C: citric acid was then added. The citric acid was used to fulfil a mole ratio of citric acid/total cations around 1.5/1. After stirring the precursor solution, ethylene glycol was added in an equal amount to the citric acid. The obtained solution was heated at 150 °C for 12 h and resulted in a polymer-like solid material. This resin was slowly heated to 300 °C and, after several sintering stages, it was finally calcined at 1400 °C for 4 h [100]. The pressed La_0.85_Sr_0.15_Ga_0.8_Mg_0.2_O_3−δ_ samples were found to be single-phase after they were obtained via the Pechini method and annealed at 1400 °C for 6 h [105].

A La_0.8_Sr_0.2_Ga_0.85_Mg_0.15_O_3−δ_ sample was also obtained via the glycine-nitrate combustion method [106]. Ga, La_2_O_3_, MgO and SrCO_3_ powders were dissolved in strong HNO_3_ and mixed with water. Glycine was then added with a molar ratio of glycine/nitrate ions equal to 1:1. The glass beaker with the precursor glycine–nitrate solution was heated on a hot plate with spontaneous burning, which resulted in a white powder. Dense samples were formed at a temperature range of 1400–1550 °C for 6 h at each stage [106]. A similar method was used in [107] for the synthesis of La_0.9_Sr_0.1_Ga_0.8_Mg_0.2_O_3−δ_. The experimental procedure included the heating of the precursor glycine–nitrate solution at 550 °C upon combustion, initial calcination of voluminous oxide powders at 800 °C for 3 h, annealing the powders at 1000 °C and final annealing at 1300 °C for 2 h. It should be noted that the authors of [107] could not achieve single-phase sample. Huang and Goodenough also concluded that a La_0.8_Sr_0.2_Ga_0.83_Mg_0.17_O_3−δ_ single-phase material cannot be formed via hydrothermal treatment synthesis [100]. A typical diagram of La_1−*x*_Sr*_x_*Ga_1−*y*_Mg*_y_*O_3−δ_ synthesis via the glycine–nitrate combustion method described in [99] is presented in Figure 5c.

In [110], Ishikawa et al., prepared La_0.9_Sr_0.1_Ga_0.8_Mg_0.2_O_3−δ_ and La_0.9_Sr_0.1_Ga_0.7_Mg_0.3_O_3−δ_ samples via self-propagating high-temperature synthesis from La_2_O_3_, SrCO_3_, Ga_2_O_3_, Mg and NaClO_4_. An initial powder mixture was supplied to a self-propagating synthesis reactor: it was then ignited with a disposable carbon foil in contact with the sample. The obtained powders were washed with water to remove NaCl. The samples were pressed into disks in vacuum and then sintered at a temperature range of 1000–1500 °C for 6 h in air. An alternative process for La_0.9_Sr_0.1_Ga_0.8_Mg_0.2_O_3−δ_ synthesis based on a preliminarily mechanically activated powder mixture was proposed by Ishikawa et al. [111]. The initial mixture was grinded in a planetary mill with stainless steel balls. The powder sample was pressed into a disk, which was placed in a self-propagating synthesis reactor: the aforementioned algorithm [110] was then used.

The literature points out that temperature of about 1400 °C (or more) is required for the synthesis of single-phase LSGM samples. Figure 5d presents the thermal evolution of the XRD pattern for a La_0.8_Sr_0.2_Ga_0.83_Mg_0.17_O_3−δ_ precursor powder [100]. The powders calcined at the intermediate temperatures were multiphase, containing La_0.8_Sr_0.2_Ga_0.83_Mg_0.17_O_3−δ_ and La_2_O_3_, LaSrGa_3_O_7_ and La_2_O_2_CO_3_ impurities. A single-phase La_0.8_Sr_0.2_Ga_0.83_Mg_0.17_O_3−δ_ sample with a cubic structure was formed during calcination at 1400 °C.

It is worth noting that the crystal structure of the obtained LSGM samples depends on the strontium and manganese dopant contents. Basic LaGaO_3_ at room temperature has an orthorhombic structure [113] but varying the doping contents can change the crystal structure symmetry [100,114]. Generally, the substitution of La^3+^-ions with Sr^2+^-ions increases the tolerance factor *t* (Equation (1)), while Ga-with-Mg substitution decreases it. Therefore, the *t* factor for La_1−*x*_Sr*_x_*Ga_1−*y*_Mg*_y_*O_3−δ_ is nearly equal to that calculated for undoped LaGaO_3_.

**Figure 6:**
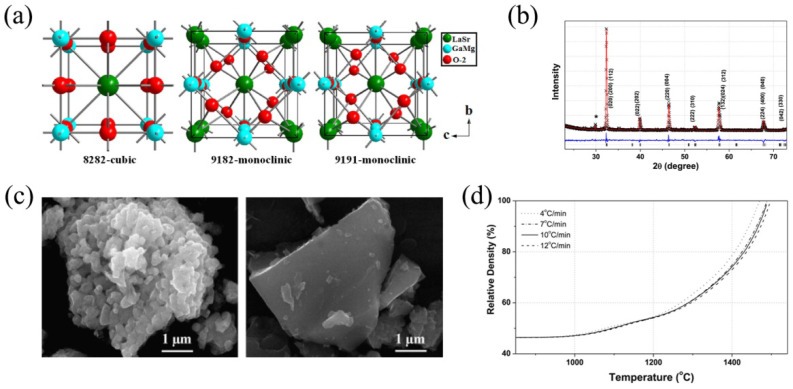
Properties of LaGaO_3_-based phases: (**a**) the crystal structure of La_0.8_Sr_0.2_Ga_0.8_Mg_0.2_O_3−δ_ (8282), La_0.9_Sr_0.1_Ga_0.8_Mg_0.2_O_3−δ_ (9182) and La_0.9_Sr_0.1_Ga_0.9_Mg_0.1_O_3−δ_ (9191). Reproduced from [114] with permission from John Wiley & Sons, Inc., 2021; (**b**) observed and Rietveld-refined XRD patterns of La_0.9_Sr_0.1_Ga_0.8_Mg_0.2_O_3−δ_. Reproduced from [115] with permission by Elsevier Ltd., 2018; (**c**) an SEM micrograph of a La_0.9_Sr_0.1_Ga_0.8_Mg_0.2_O_3−δ_ ceramic obtained via mechanically activated and conventional self-propagating synthesis. Reproduced from [111] with permission by Elsevier Ltd., 2009; (**d**) the temperature dependencies of the relative density of a La_0.9_Sr_0.1_Ga_0.8_Mg_0.2_O_3−δ_ ceramic material. Reproduced from [115] with permission from Elsevier Ltd., 2018.

The *t* factor is equal to 1 for La_0.8_Sr_0.2_Ga_0.8_Mg_0.2_O_3−δ_, which exhibits an ideal *Pm-3m* cubic structure with a unit cell parameter of *a* = 3.9146(1) Å [114] (Figure 6a). According to [114], the crystal structure of La_0.9_Sr_0.1_Ga_0.8_Mg_0.2_O_3−δ_ and La_0.9_Sr_0.1_Ga_0.9_Mg_0.1_O_3−δ_ samples (Figure 5a) was refined in a *I2/a* monoclinic space group.

The crystal structure of LaGaO_3_ and La_0.9_Sr_0.1_Ga_0.8_Mg_0.2_O_3−δ_ samples was investigated via powder neutron diffraction at 25, 800 and 1000 °C in [116]. According to the Rietveld refinement analysis of the diffraction data collected at 25 °C, an orthorhombic structure was observed for both samples: fitting was provided in the *Pnma* space group for LaGaO_3_ (unit cell parameters were equal to *a* = 5.4908(1), *b* = 7.7925(1) and *c* = 5.5227(1) Å) and in the *Imma* space group for La_0.9_Sr_0.1_Ga_0.8_Mg_0.2_O_3−δ_ (unit cell parameters were equal to *a* = 5.5179(1), *b* = 7.8200(1) and *c* = 5.5394(1) Å). The high temperature measurements [116] show that the LaGaO_3_ sample possessed a rhombohedral structure in the *R-3c* space group (unit cell parameters were equal to *a* = 5.5899(1) Å and *a* = 5.5987(1) Å at 800 and 1000 °C, correspondingly), whereas La_0.9_Sr_0.1_Ga_0.8_Mg_0.2_O_3−δ_ exhibits a cubic structure in the *Pm3m* space group (unit cell parameters were equal to *a* = 3.9760(1) Å and *a* = 3.9866(1) Å at 800 and 1000 °C, correspondingly). Similar data at 25 °C (the *Imma* space group*, a* = 5.5056(9), *b* = 7.8241(7), *c* = 5.5387(5) Å) for a La_0.9_Sr_0.1_Ga_0.8_Mg_0.2_O_3−δ_ sample obtained via solid-state route and sintered at 1350 °C for 2 h was reported in [115]. However, this sample consisted of an LSGM phase and a LaSrGa_3_O_7_ impurity phase, as indicated by ‘*’ in Figure 6b. This fact proves the necessity of sintering temperatures of 1400 °C for obtaining single-phase LSGM samples.

Comparative analysis of the microstructural parameters for La_0.9_Sr_0.1_Ga_0.8_Mg_0.2_O_3−δ_ disks sintered at 1400 °C for 6 h obtained via the self-propagating high-temperature and solid-reaction synthesis techniques showed that the first sample was denser [110]. The relative densities of the samples were 98 and 92%, respectively, despite the fact that the sintering temperature for the first disk was 100 °C lower than that for the second one. Images in Figure 6c show the SEM micrographs of La_0.9_Sr_0.1_Ga_0.8_Mg_0.2_O_3−δ_ samples obtained via self-propagating synthesis with and without mechanical activation of the starting mixture for 24 h [111]. These SEM images testify that mechanically activated self-propagating synthesis provided the high-grade powders with nano-size particles. The specific surface areas of the samples were 3.36 and 2.06 m^2^ g^−1^, respectively. Based on both studies, Ishikawa et al. [110,111] concluded that this proved the advantages of using self-propagating high-temperature synthesis (especially with mechanical activation of the starting mixture) in comparison with the solid-reaction method.

The evolution of a La_0.9_Sr_0.1_Ga_0.8_Mg_0.2_O_3−δ_ sample’s density against temperature was provided in by Batista et al. [115]. Based on dilatometry experimental results (Figure 6d), the authors separated the process into three steps: an insignificant increase of relative density at 25–1000 °C; gradual densification at 1000–1300 °C; and, finally, a fast densification above 1300 °C. According to [117], a relative density of over 99% was achieved after calcination at 1450 °C for 6 h.

Summing up the review section, which was devoted to the synthesis methods of Sr, Mg-doped LaGaO_3_ oxides as electrolyte materials, the self-propagating high-temperature synthesis with mechanical activation of the starting mixtures can be identified as one of the most optimal techniques. The above-mentioned method can obtain the single-phase La_0.9_Sr_0.1_Ga_0.8_Mg_0.2_O_3−δ_ powders with high specific surface areas, a narrow distribution of nano-size particles, and high relative densities for the sintered ceramic samples.

### 3.2. Functional Properties

**Figure 7:**
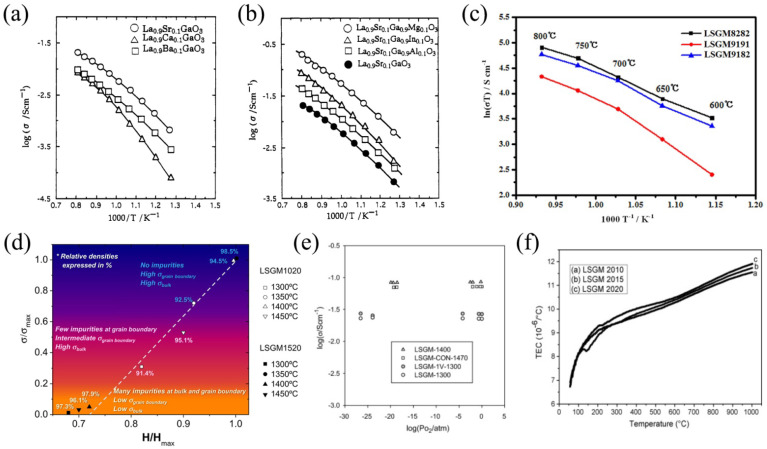
Properties of LaGaO_3_-based phases: (**a**) the crystal structure of La_0.8_Sr_0.2_Ga_0.8_Mg_0.2_O_3−δ_ (8282), La_0.9_Sr_0.1_Ga_0.8_Mg_0.2_O_3−δ_ (9182) and La_0.9_Sr_0.1_Ga_0.9_Mg_0.1_O_3−δ_ (9191). Reproduced from [114] with permission from John Wiley & Sons, Inc., 2021; (**b**) observed and Rietveld-refined XRD patterns of La_0.9_Sr_0.1_Ga_0.8_Mg_0.2_O_3−δ_. Reproduced from [115] with permission form Elsevier Ltd., 2018; (**c**) an SEM micrograph of a La_0.9_Sr_0.1_Ga_0.8_Mg_0.2_O_3−δ_ ceramic obtained via mechanically activated and conventional self-propagating synthesis. Reproduced from [111] with permission by Elsevier Ltd., 2009; (**d**) the temperature dependencies of the relative density of a La_0.9_Sr_0.1_Ga_0.8_Mg_0.2_O_3−δ_ ceramic material. Reproduced from [115] with permission from Elsevier Ltd., 2018; (**e**) conductivity of La_0.8_Sr_0.2_Ga_0.8_Mg_0.2_O_3−δ_ as a function of oxygen partial pressure. Reproduced from [109] with permission from Elsevier Ltd., 2011; (**f**) the temperature dependencies of TEC for La_0.8_Sr_0.2_Ga_0.9_Mg_0.1_O_3−δ_ (LSGM2010), La_0.8_Sr_0.2_Ga_0.85_Mg_0.15_O_3−δ_ (LSGM2015) and La_0.8_Sr_0.2_Ga_0.8_Mg_0.2_O_3−δ_ (LSGM2020). Reproduced from [121] with permission from Elsevier Ltd., 2009.

In 1994, Ishihara et al. [51] were the first to show that the La-substitution of LaGaO_3_ with strontium and gallium with magnesium increased the electrical conductivity of doped materials (Figure 7a,b) owing to the formation of oxygen vacancies in La_1−*x*_Sr*_x_*Ga_1−*y*_Mg*_y_*O_3−δ_ [118].

**Figure A2:**
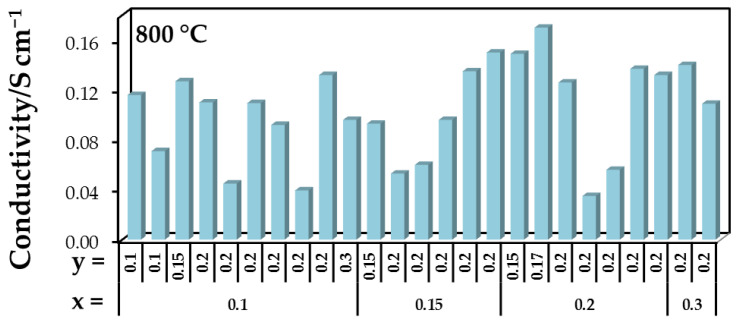
Total conductivity of theLa_1−*x*_Sr*_x_*Ga_1−*y*_MgyO_3−δ_ ceramic materials at 800 °C. These data are taken from Table 2.

The measurements of Ishihara [51], Stevenson [119] and Goodenough [95] demonstrate that the La_1−*x*_Sr*_x_*Ga_1−*y*_Mg*_y_*O_3−δ_ samples possess maximal electrical conductivity values at *x* = 0.15/0.2 and *y* = 0.2, as can be seen in Table 2. It should be also noted that conductivity of nominally similar materials can be varied over a wide range (see Figure A2). This confirms that the microstructural parameters of ceramics, as well as the presence of insulating impurity phases, considerably affect the transport properties of gallates, encouraging the continuous search for their new synthesis and fabricating techniques.

Hayashi et al. [120] concluded that the electrical conductivity of La_1−*x*_Sr*_x_*Ga_1−*y*_Mg*_y_*O_3−δ_ becomes greater when approaching the tolerance factor of the doped sample to *t* for LaGaO_3_ and decreases when the tolerance factor for the doped samples differed from *t* for LaGaO_3_. It was established that increasing the Sr, Mg-doping levels led to the association of oxygen vacancies [51,119,120]; for this reason, further electrical investigations of the doped-LaGaO_3_ oxides were performed on La_1−*x*_Sr*_x_*Ga_1−*y*_Mg*_y_*O_3−δ_ samples with a fixed content of Sr and Mg dopants (nearly 20 mol.%, i.e., *x* = *y* = 0.2). The literature on the transport properties of La_1−*x*_Sr*_x_*Ga_1−*y*_Mg*_y_*O_3−δ_ ceramic samples is summarised in Table 2. Figure 7c presents the temperature dependencies of conductivity for the La_0.9_Sr_0.1_Ga_0.9_Mg_0.1_O_3−δ_ (LSGM9191), La_0.9_Sr_0.1_Ga_0.8_Mg_0.2_O_3−δ_ (LSGM9182) and La_0.8_Sr_0.2_Ga_0.8_Mg_0.2_O_3−δ_ (LSGM8282) samples obtained in [114]. These data agree with the conclusion that the maximal conductivity for LSGM is achieved at *x* = *y* = 0.2.

It was shown in [119] that the ion-transfer numbers were nearly equal to 1. For La_0.9_Sr_0.1_Ga_0.8_Mg_0.2_O_3−δ_ and La_0.8_Sr_0.2_Ga_0.8_Mg_0.2_O_3−δ_ ceramic samples, the oxygen-ion transference numbers were found to be equal 1 at 700–1000 °C [107], confirming the presence of electrolyte-type behaviour. Savioli and Watson [134] studied the defect structure of LaGaO_3_ upon the use of various doping strategies using DFT calculations. They confirmed that Sr-, Ba-, and Mg-doping should result in the greatest improvements to the ionic conductivity of the LaGaO_3_ parent phase, while the Ni^2+^-, Co^2+^-, Fe^2+^-, and Zn^2+^-doping is responsible for the generation of a mixed ionic-electronic conducting behaviour. Sr- and Mg- co-doped LaGaO_3_ complex oxides are predominantly oxygen-ionic conductors, for which the electronic conductivity levels are 3–4 magnitudes lower compared to the oxygen-ionic conductivity levels [135].

According to [125], the dependence ln(*σT*) vs. 1/*T* had a break at 700 °C for La_0.85_Sr_0.15_Ga_0.8_Mg_0.2_O_3−δ_, which indicates that the activation energy value of oxygen-ion conductivity at a low-temperature range was higher than that at a high-temperature range.

A linear correlation between hardness and total ionic conductivity was revealed in [126] for La_0.9_Sr_0.1_Ga_0.8_Mg_0.2_O_3−δ_ and La_0.85_Sr_0.15_Ga_0.8_Mg_0.2_O_3−δ_ samples. It was shown that the electrical and mechanical properties of La_1−*x*_Sr*_x_*Ga_1−*y*_Mg*_y_*O_3−δ_ are strongly defined by microstructural peculiarities and the presence of low-conductive LaSrGaO_4_ and LaSrGa_3_O_7_ impurity phases [123]. The LaSrGaO_4_ phase exhibits a tetragonal structure K_2_NiF_4_-type and crystalizes in the *I4/mmm* space group; its conductivity is found to be around 2·10^−7^ S cm^−1^ at 900 °C [136]. The LaSrGa_3_O_7_phase belongs to a melilitestructure described in the *P421m* space group; its ionic conductivity level is around 2·10^−6^ S cm^−1^ at 800 °C [137]. The maximum values of ionic conductivity and hardness were achieved for single-phase La_0.9_Sr_0.1_Ga_0.8_Mg_0.2_O_3−δ_ (LSGM1020) and La_0.85_Sr_0.15_Ga_0.8_Mg_0.2_O_3−δ_ (LSGM1520) samples with a high relative density, as shown in Figure 7d. With a significant amount of impurity phases at the grain boundaries, the samples exhibited a gradual decrease in hardness and the grain boundary conductivity, which resulted in a decreasing total conductivity. The data in Table 2 may also be analysed from the aforementioned perspective.

The electrical conductivity of La_0.8_Sr_0.2_Ga_0.8_Mg_0.2_O_3−δ_ was investigated over a *Po*_2_ range of 10^−27^–1 atm at 700 °C [109]. The results of the measurements are presented in Figure 7e for La_0.8_Sr_0.2_Ga_0.8_Mg_0.2_O_3−δ_ samples, sintered at 1470 °C (LSGM-CON-1400), 1400 °C (LSGM-1400) and 1300 °C (LSGM-1300), and an LSGM sample with 1 wt% V_2_O_5_ sintered at 1300 °C (LSGM-1V-1300). All these samples show an approximately constant conductivity over the measuring pO_2_ range, implying a realization of the electrolytic conduction behaviour.

The thermal expansion of La_1−*x*_Sr*_x_*Ga_1−*y*_Mg*_y_*O_3−δ_ was studied by Baskaran et al. [138]. The TEC values measured for the La_0.9_Sr_0.1_Ga_0.8_Mg_0.2_O_3−δ_ sample were equal to 10 × 10^−6^ K^−1^ over a low-temperature range and 13.5–14.0 × 10^−6^ K^−1^ above 600 °C. Lee et al. [99] reported about an average TEC of 12.1 × 10^−6^ K^−1^ for La_0.8_Sr_0.2_Ga_0.8_Mg_0.2_O_3−δ_ at a temperature range of 25–1000 °C, which is close to 12.3 × 10^−6^ K^−1^ for a La_0.65_Sr_0.3_MnO_3−δ_ electrode at the same temperatures [92].

The expansion behaviour for La_1−*x*_Sr*_x_*Ga_1−*y*_Mg*_y_*O_3−δ_ is correlated with its crystal structure in the observed temperature range. Therefore, the presence of a phase transition from an orthorhombic phase to a cubic one for La_0.9_Sr_0.1_Ga_0.8_Mg_0.2_O_3−δ_ [116] and the existence of an ideal perovskite cubic structure for La_0.8_Sr_0.2_Ga_0.8_Mg_0.2_O_3−δ_ [114] are responsible for the aforementioned variations in their thermal expansion behaviour.

Datta et al. [121] observed that the temperature of phase transition from an orthorhombic to a rhombohedral structure for La_1−*x*_Sr*_x_*Ga_1−*y*_Mg*_y_*O_3−δ_ increased as Mg content increased at a fixed Sr content, as shown in Figure 7f, and decreased with increasing Sr content at a fixed Mg content. The effect of Sr and Mg co-doping on TEC values was explained for La_1−*x*_Sr*_x_*Ga_1−*y*_Mg*_y_*O_3−δ_ in terms of the amount of generated oxygen vacancies. It was concluded that TEC values increased as oxygen vacancies increase, regardless of the dopant type. This was the result of the binding energy weakening as a result of oxygen vacancy formation.

Shkerin et al. [139] analysed the structure and phase transitions of La_0.88_Sr_0.12_Ga_0.82_Mg_0.18_O_3−δ_ using dilatometry, XRD and Raman spectroscopy. According to the obtained data, La_0.88_Sr_0.12_Ga_0.82_Mg_0.18_O_3−δ_ exhibited two phase transitions of the second order at 502 and 607 °C. The first transition was attributed to a phase transition from an orthorhombic phase to a cubic one, while the second phase transition was attributed to the ordering of the oxygen vacancies.

Wu et al. [140] studied transport properties of La_0.85_Sr_0.15_Ga_0.8_Mg_0.2_O_3−δ_ upon the partial or full Sr-substitution with calcium or barium. Their analyses have shown that both types of substitution result in a decrease in ionic conductivity by 20–30%. However, at the same time, the Ca-substituted ceramic materials showed higher conductivities compared to the Ba-substituted analogues. This confirms that strontium is an ideal dopant (from the steric and energetic viewpoints) to be introduced into the La-sublattice of LaGaO_3_-based phases.

The chemical compatibility of La_1−*x*_Sr*_x_*Ga_1−*y*_Mg*_y_*O_3−δ_ was investigated with oxide materials used in SOFCs, cathodes [141,142,143,144,145,146,147,148,149,150,151,152] and anodes [153,154,155,156,157,158,159,160,161,162,163,164,165,166,167]: this is presented in the corresponding reviews [28,56,153].

Chemical interactions between a La_0.9_Sr_0.1_Ga_0.8_Mg_0.2_O_3−δ_ electrolyte and cathode materials such as La_0.65_Sr_0.3_MnO_3−δ_, La_0.7_Sr_0.3_CoO_3−δ_, La_0.65_Sr_0.3_FeO_3−δ_, La_0.65_Sr_0.3_NiO_3−δ_ and La_0.6_Sr_0.4_Co_0.2_Fe_0.8_O_3−δ_ are demonstrated in [141]. The LSGM/cathode powders were mixed at a weight ratio of 1:1, pressed into disks and annealed at 1300 °C for 3 h in air. The XRD data revealed that impurity phases were not formed in the LSGM mixed with La_0.65_Sr_0.3_MnO_3−δ_, La_0.7_Sr_0.3_CoO_3−δ_, and La_0.65_Sr_0.3_FeO_3−δ_, but appear in the calcined mixtures with La_0.65_Sr_0.3_NiO_3−δ_ and La_0.6_Sr_0.4_Co_0.2_Fe_0.8_O_3−δ_. The absence of reactivity between La_0.8_Sr_0.2_Ga_0.8_Mg_0.2_O_3−δ_ and La_0.8_Sr_0.2_MnO_3−δ_ was also confirmed during calcination at 800 °C [142].

Sydyknazar et al. [143] showed that La_0.83_Sr_0.17_Ga_0.8_Mg_0.2_O_3−δ_ exhibited good chemical compatibility with a novel cathode material, Sr_0.9_Ba_0.1_Co_0.95_Ru_0.05_O_3−δ_, after joint calcination at 1100 °C for 12 h. According to the literature, La_0.9_Sr_0.1_Ga_0.8_Mg_0.2_O_3−δ_ does not react with the following cathodes: La_0.4_Sr_0.6_Co_0.9_Sb_0.1_O_3−δ_ after heat treatment at 1150 °C for 6 h [144], SrCo_0.8_Fe_0.1_Nb_0.1_O_3−δ_ at 950 °C for 10 h [145], BaCo_0.7_Fe_0.2_Ta_0.1_O_3−δ_ at 950 °C for 10 h [146] and Sr_2_Ti_0.8_Co_0.2_FeO_6−δ_ after at 950 °C for 10 h [147]. According to Tarancón et al. [148], La_0.8_Sr_0.2_Ga_0.8_Mg_0.2_O_3−δ_ interacted with a GdBaCo_2_O_5+δ_ cathode at temperatures above 900 °C, forming BaLaGa_3_O_4_ and BaLaGa_3_O_7_ secondary phases.

An analysis of works devoted to Ruddlesden–Popper phases demonstrates that La_0.9_Sr_0.1_Ga_0.8_Mg_0.2_O_3−δ_ and Pr_2__−_*_x_*La*_x_*Ni_0.85_Cu_0.1_Al_0.05_O_4+δ_ (*x* = 0, 0.2, 0.5, 1.0) have no interactions at 1000 °C for 5 h [149], but La_0.95_Sr_0.05_Ga_0.9_Mg_0.1_O_3−δ_ reacted with Nd_2_NiO_4+δ_ after annealing at 1000 °C for 5 h [150]. Equally, La_0.85_Sr_0.15_Ga_0.85_Mg_0.15_O_3−δ_ reacted with Pr_2−*x*_Ca*_x_*NiO_4+δ_ after annealing at 900 °C for 10 h (*x* = 0, 0.5) [151] and at 1200 °C for 1 h (*x* = 0, 0.3) [152].

Zhang et al. [154] showed that a La_0.9_Sr_0.1_Ga_0.8_Mg_0.2_O_3−δ_ electrolyte reacted with the nickel component in a Ni-SDC anode. The chemical interaction between LSGM and the composite was due to the interface diffusion of nickel from the anode to the LSGM electrolyte; this led to the formation of La-based poor-conductive secondary phases, which block oxygen-ion transport. The unit cell design with a buffer layer of SDC was suggested as an effective way of avoiding the problem of interface diffusion [155]. However, chemical reactivity was observed between La_1−*x*_Sr*_x_*Ga_1−*y*_Mg*_y_*O_3−δ_ and buffer layers of Gd_0.1_Ce_0.9_O_1.95_, scandia-doped zirconia [156] and Gd_0.8_Ce_0.2_O_1.9_ [157].

An alternate solution to the problem of nickel interface diffusion from a Ni-based anode is to find novel anode materials. A study of the chemical compatibility between La_0.9_Sr_0.1_Ga_0.8_Mg_0.2_O_3−δ_ and Fe_2_O_3_, Co_2_O_3_, NiO as anode materials is provided in [158]. Powder mixtures of LSGM with metal oxides at a weight ratio of 1:1 were mixed in ethanol, pressed into pellets and annealed at 1150, 1250 and 1350 °C for 2 h. The obtained XRD data showed that the LSGM reacted with NiO and Co_2_O_3_ at 1150 °C, while a detectable reaction with Fe_2_O_3_ occurred only after calcination at 1350 °C.

Du and Sammes [159] reported good chemical compatibility between La_0.8_Sr_0.2_Ga_0.8_Mg_0.2_O_3−δ_ and an alternative La_0.75_Sr_0.25_Cr_0.5_Mn_0.5_O_3_ anode at a temperature range of 1100–1500 °C. However, the authors note that a low-conductivity phase formed if the annealing time was more than 6 h or the annealing temperature was greater than 1500 °C.

Good chemical compatibility between LSGM and anodes with a double perovskite structure was shown for: La_0.9_Sr_0.1_Ga_0.8_Mg_0.2_O_3−δ_ and Sr_2_TiMoO_6−δ_ after calcining the samples at 1000 °C for 10 h in an atmosphere of 5% H_2_/Ar [160], La_0.8_Sr_0.2_Ga_0.8_Mg_0.2_O_3−δ_ and Sr_2_Fe_1.5_Mo_0.5_O_6−δ_ after heat treatment at 1200 °C for 24 h in air [161], La_0.88_Sr_0.12_Ga_0.82_Mg_0.18_O_3−δ_ with Sr_2_NiMoO_6−δ_ at 1000 °C for 20 h [162,163] and Sr_2_Ni_0.75_Mg_0.25_MoO_6−δ_ at 1100 °C for 20 h [164] and at 1250 °C for 2 h [163]. The formation of secondary phases between LSGM and double perovskite anodes was observed for La_0.9_Sr_0.1_Ga_0.8_Mg_0.2_O_3−δ_ and Sr_2_MgMoO_6−δ_ after calcining at 1100 °C [165], for La_0.88_Sr_0.12_Ga_0.82_Mg_0.18_O_3−δ_ and Sr_2_ZnMoO_6_ at 1000 °C for 20 h [166] and for La_0.8_Sr_0.2_Ga_0.8_Mg_0.2_O_3−δ_ at 1300 °C for 10 h with Sr_2_Ni_0.7_Mg_0.3_MoO_6−δ_ [167] and, after heat treatment at 1200 °C for 24 h, with Sr_2_CoMoO_6−δ_ [161], Sr_2_NiMoO_6−δ_ [161] and Sr_2_MgMoO_6−δ_ [168].

According to Takano et al. [165], La_0.9_Sr_0.1_Ga_0.8_Mg_0.2_O_3−δ_ did not react with Ce_0.8_La_0.2_O_1.8_ after annealing at 1300 °C for 1 h; therefore, it was concluded that La_0.9_Sr_0.1_Ga_0.8_Mg_0.2_O_3−δ_ and Ce_0.8_La_0.2_O_2−δ_ might be recommended as SOFC electrolyte and buffer materials, respectively, with Sr_2_MgMoO_6−δ_ used as the anode material. However, a comprehensive investigation of the chemical compatibility between various compositions of La_1−*x*_Sr*_x_*Ga_1−*y*_Mg*_y_*O_3−δ_ and lanthanum-doped CeO_2_, provided in [169], showed that only a La_0.9_Sr_0.1_Ga_0.8_Mg_0.2_O_3−δ_/Ce_0.6_La_0.4_O_2−δ_ mixture did not result in additional phases after being annealed twice at 1350 °C for 2 h at each stage.

### 3.3. Applications in SOFCs

The problem of reactivity between the LSGM and SOFC electrode materials during sintering can be solved by reducing sintering temperatures or/and using the SDC buffer layer as a barrier, eliminating lanthanum- and nickel-cation diffusion. Several unit cell designs have been proposed in the literature. Table 3 presents a summary of electrochemical performances for different types of hydrogen-fuelled SOFCs with LSGM-based electrolytes. These data testify that enhanced power densities were achieved for electrolyte-supported SOFCs when the LSGM electrolyte thickness was in a range of 100–300 μm. Buffer layers of doped ceria were used between the electrolyte and anode: Ce_0.8_Sm_0.2_O_2−δ_ [144,145,149,155,160,167], Ce_0.8_Gd_0.2_O_2−δ_ [170] and Ce_0.6_La_0.4_O_2−δ_ [171,172].

**Figure A3:**
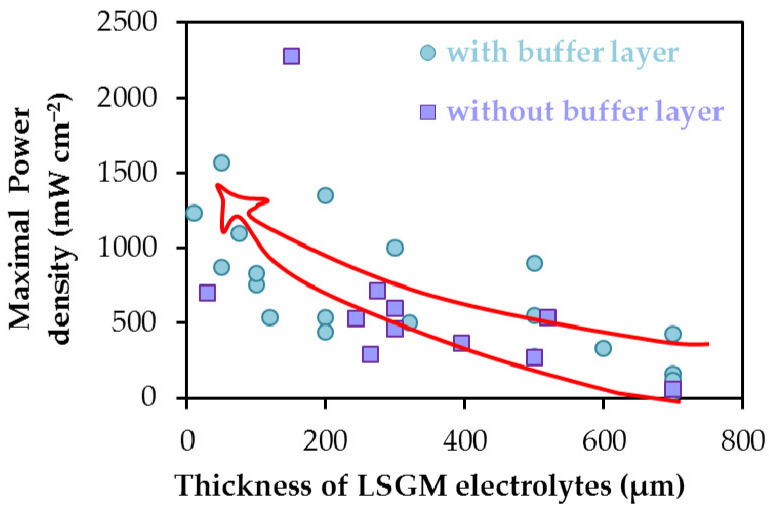
Maximum power densities of SOFCs based on the LSGM-based electrolytes at 800 °C. These data are taken from Table 3.

Considering the details in Figure A3, one can see that the SOFCs’ power density tends to increase with a decrease in the electrolyte’s thickness (due to a corresponding decline in the ohmic resistance) despite the existence/absence of CeO_2_-based buffer layers. Nevertheless, the performance of the compared SOFCs varies greatly, even for close electrolyte thicknesses, indicating that other functional components (cermets, oxygen electrodes) have a significant effect on the achievable output characteristics.

**Figure 8:**
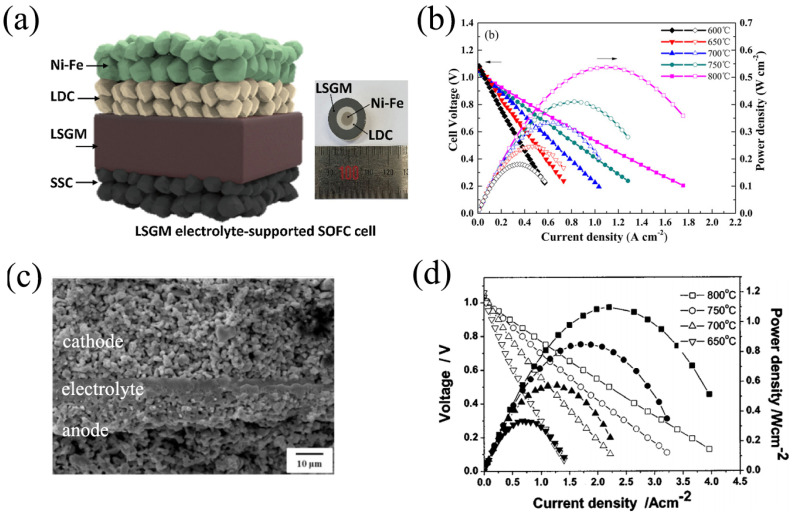
Design and performances of LaGaO_3_-based SOFCs: (**a**) schematic illustration of Ni–Fe/Ce_0.6_La_0.4_O_2−δ_/La_0.9_Sr_0.1_Ga_0.8_Mg_0.2_O_3−δ_/Sm_0.5_Sr_0.5_O_3−δ_. Reproduced from [171] with permission from Elsevier Ltd., 2021; (**b**) *I*–*V* and power density curves of the electrolyte-supported cell with an LSGM electrolyte at different temperatures. Reproduced from [170] with permission from John Wiley & Sons, Inc., 2018; (**c**) SEM micrograph of an anode-supported cell with an LSGM electrolyte. Reproduced from [174] with permission from Elsevier Ltd., 2002; (**d**) *I*–*V* and power density curves of an anode-supported cell with a Ce_0.6_La_0.4_O_1.8_-LSGM bi-layered electrolyte at different temperatures. Reproduced from [178] with permission from The Electrochemical Society, 2004.

A diagram of a typical LSGM-supported cell with a barrier layer between the anode and the electrolyte, using a Ni-Fe/Ce_0.6_La_0.4_O_2−δ_/La_0.9_Sr_0.1_Ga_0.8_Mg_0.2_O_3−δ_/Sm_0.5_Sr_0.5_O_3−δ_ cell, is presented in Figure 8a. In [171], it was shown that the OCV values were equal to 1.07 and 1.15 V at 800 °C and 700 °C, respectively, and there was no significant difference in the thickness of the Ce_0.6_La_0.4_O_1.8_ interlayer. This LSGM-supported cell yielded up to 2200 and 1350 mW cm^−2^ at 850 and 800 °C, respectively. The typical *I*–*V* curve and power densities at different temperatures for the LSGM-supported cell are shown in Figure 8b, which is based on the Ni-Ce_0.8_Gd_0.2_O_2−δ_/Ce_0.8_Gd_0.2_O_2−δ_/(La_0.9_Sr_0.1_)_0.97_Ga_0.9_Mg_0.1_O_3−δ_/La_0.6_Sr_0.4_Fe_0.8_Co_0.2_O_3−δ_ cell tested in [170]. The maximum power density of the aforementioned cell reached 540 mW cm^−2^ at 800 °C, while the maximum power density of a cell containing a La_0.9_Sr_0.1_Ga_0.9_Mg_0.1_O_2.9_ electrolyte reached 450 mW cm^−2^ at 800 °C. The electrode polarization resistance values of the La_0.9_Sr_0.1_Ga_0.9_Mg_0.1_O_3−δ_ and (La_0.9_Sr_0.1_)_0.97_Ga_0.9_Mg_0.1_O_3−δ_ based cells were equal to 0.34 and 0.30 Ω cm^2^ at 800 °C, respectively.

Table 3 shows that, for electrode-supported SOFCs with thin-film LSGM electrolytes, a barrier layer between the electrolyte and the electrodes is not necessary [174,175,176,184,185]. An anode-supported cell containing a La_0.9_Sr_0.1_Ga_0.8_Mg_0.2_O_3−δ_ film deposited on an anode supported substrate using radio-frequency magnetron sputtering was fabricated in [174]. The anode substrate was composed of a Ni-Sm_0.2_Ce_0.8_O_2−δ_ functional layer and a Ni collector layer; an LSGM-La_0.6_Sr_0.4_Co_0.2_Fe_0.8_O_3−δ_ composite layer was used as a cathode. The obtained SOFC revealed no cracking, delamination or discontinuity, as shown in Figure 8c. The polarization resistance of an anode-supported cell containing a La_0.9_Sr_0.1_Ga_0.8_Mg_0.2_O_3−δ_ film decreased from 0.41 to 0.05 Ω cm^2^ as the temperature increased from 600 to 800 °C. The OCV and *P*_max_ values were in the range of 0.85–0.95 V and 650-1420 mW cm^−2^, respectively, at a temperature range of 600–750 °C.

Combining the two approaches for SOFC design can be found in [178,179,180,181]. Bi et al. deposited a Ce_0.6_La_0.4_O_2−δ_/LSGM bi-layer film on a Ni-Ce_0.9_Gd_0.1_O_2−δ_ anode. Therefore, the cell design allowed for high OCVs (1.02 and 1.043 V at 800 °C) and high power density values (1100 and 1565 mW cm^−2^ at 800 °C) to be achieved at a LDC/LSGM bi-layer thickness of 100 and 65 μm, respectively [178,179]. The *I*–*V* and power density curves for a Ni-Ce_0.6_La_0.4_O_2−δ_/Ce_0.6_La_0.4_O_2−δ_/LSGM(100 μm)/La_0.9_Sr_0.1_O_3−δ_-Ce_0.55_La_0.45_O_2−δ_ cell at different temperatures, are shown in Figure 8d [178]. Ju et al. [181] reached a paramount performance of 1790 mW cm^−2^ at 700 °C for a SOFC based on an LSGM film with a thickness of 6 μm: this used an SDC buffer layer with a thickness of 500 nm, which was deposited on a Ni–Fe porous anode support. After a thermal cycle going from 700 to 25 °C, the fabricated cell showed an OCV of 1.1 V and *P*_max_ of 1620 mW cm^−2^, which was almost the same as the first cycles.

According to a number of investigations [179,182,183,198], the most effective design for SOFCs composed of barrier layers is the LDC/LSGM/LDC tri-layered electrolyte. Bi et al. reported [179] that an anode-supported SOFC with an LDC/LSGM/LDC tri-layered electrolyte film significantly increased when using a cell with an LDC/LSGM bi-layered electrolyte film with the same thickness [178]. Guo et al. [183], depositing an LDC/LSGM/LDC tri-layer with thickness of 30 μm on a Ni-Ce_0.8_Sm_0.2_O_2−δ_ anode, fabricated a cell with a 75 mL min^−1^ H_2_ flow rate that generated 1230 W cm^−2^ at 800 °C. The specific ohmic resistance across the LDC/LSGM/LDC tri-layer electrolyte film was measured to be equal to 0.086 Ω cm^2^ at 800 °C. The obtained data showed that the polarization resistance was higher than the ohmic resistance at temperatures below 700 °C. A long-term stability experiment was performed on the aforementioned cell with a current density of 1000 mA cm^−2^ and a 30 mL min^−1^ H_2_ flow rate at 800 °C. The results of 95 h-test demonstrated that the maximum power density values decreased from 1.08 to 0.81 W cm^−2^. The authors of [183] suggest that there was little diffusion of the transition metal from the electrodes to the electrolyte during the test.

Serious efforts have been made to replace traditional cermet anodes with single-phase oxide materials: this is in an attempt to avoid chemical interactions. Complex oxides with double perovskite (Sr_2_MMoO_6−δ_ (M = Mg, Ti, Ni, Fe) [160,162,167,168,186,187,191,196]), layered [190,193] and perovskite [172,189] structures were successfully tested as alternative anode materials for SOFCs with LSGM electrolytes. A buffer layer of doped ceria was used to avoid chemical interactions between an LSGM electrolyte and double perovskites [160,167,168], as well as between an LSGM electrolyte and an oxide cathode [163,187,199]. The composite electrodes Sr_2_Fe_1.5_Mo_0.5_O_6−δ_-La_0.9_Sr_0.1_Ga_0.8_Mg_0.2_O_3−δ_ [191], Sr_2_CoMoO_6−δ_-La_0.9_Sr_0.1_Ga_0.8_Mg_0.2_O_3−δ_ and Sr_2_Co_0.9_Mn_0.1_NbO_6−δ_-La_0.9_Sr_0.1_Ga_0.8_Mg_0.2_O_3−δ_ [200] have been proposed to solve the thermo-mechanical incompatibility between an electrolyte and an electrode due to a mismatch in the materials’ thermal expansion [174,182,183,184,191,200,201,202,203].

An analysis of recent studies illustrates that LSGM can be used as a base matrix for the formation of both composite electrodes and new composite electrolytes [200,204,205,206,207,208,209,210]. Xu et al. [200] fabricated a cell based on a La_0.9_Sr_0.1_Ga_0.8_Mg_0.2_O_3−δ_-Ce_0.8_Gd_0.2_O_1.9_ electrolyte, with Sr_2_CoMoO_6−δ_-La_0.9_Sr_0.1_Ga_0.8_Mg_0.2_O_3−δ_ as the anode and Sr_2_Co_0.9_Mn_0.1_NbO_6−δ_-La_0.9_Sr_0.1_Ga_0.8_Mg_0.2_O_3−δ_ as the cathode. For this cell, obtained with a 95 wt.% La_0.9_Sr_0.1_Ga_0.8_Mg_0.2_O_3−δ_-5 wt.% Ce_0.8_Gd_0.2_O_2−δ_ electrolyte, the OCV, *P*_max_ and current density values at 800 °C were equal to 1.08 V, 192 mW cm^−2^_,_ and 720 mA cm^−2^, respectively [200].

The electrochemical investigations in [211,212,213,214,215] for LSGM-based SOFCs confirm that these cells can operate in both fuel cell and electrolysis cell modes. Reversible cells were fabricated in [215] with NiO–YSZ-substrate as an anode, La_0.9_Sr_0.1_Ga_0.8_Mg_0.2_O_3−δ_ film as an electrolyte and Sm_0.5_Sr_0.5_CoO_3−δ_ as an air electrode. It was established that the infiltration of cerium nitrate into the substrate was an effective means of increasing cell performance. The maximum power density of this cell at 3 M Ce nitrate infiltration achieved 950 mW cm^−2^ at 600 °C.

## 4. Conclusions

Complex oxides based on LaGaO_3_ offer a convenient basis for the design of oxygen-conducting electrolytes that can be employed in intermediate-temperature solid oxide fuel cells (SOFCs). A rational combination of appropriate dopants incorporated at various sublattices of LaGaO_3_ allows superior transport properties to be achieved for co-doped derivatives (La_1−*x*_Sr*_x_*Ga_1−*y*_Mg*_y_*O_3−δ_, LSGM). LSGM materials are considered one of the most conductive oxygen-ionic electrolytes, enabling a decrease in SOFC operation temperatures by 100–300 °C compared to YSZ-based SOFCs. As a result, very high SOFC performances (from 0.5 to 1.5 W cm^−2^ at 700 °C) were reported for lab-type electrochemical cells. However, to efficiently place laboratory studies on a manufacturing scale, several issues remain, including the development of simple and low-cost technologies for electrolyte preparation (including thin-film forms), searching for strategies to improve the chemical stability of LSGM with other SOFC components (especially with nickel) and the design of new electrochemically active electrodes. In this regard, the present review serves as the starting point for further research in fields such as solid-state chemistry, physical chemistry, electrochemistry and the technology of LaGaO_3_-based materials and electrochemical cells.

## Figures and Tables

**Table 1:** Total conductivity and activation energy values for LaAlO_3_ ceramic materials. Figure A1 (see the Appendix A) provides a visualization of these data.

Sample	*T* (°C)	*σ* (S cm^−1^)	*E_a_* (eV)	Ref.
LaAlO_3_	900	6 × 10^−4^	1.30	[53]
LaAlO_3_	700	6.7 × 10^−4^	0.99	[71]
LaAlO_3_	900	1.1 × 10^−6^	1.83	[75]
LaAlO_3_	900	1.4 × 10^−3^	1.88	[80]
LaAlO_3_	800	2.0 × 10^−4^	1.30	[83]
La_0.9_Ca_0.1_AlO_3−δ_	900	6.0 × 10^−3^	1.08	[65]
La_0.9_Sr_0.1_AlO_3−δ_	900	1.1 × 10^−2^	1.14	[80]
La_0.9_Sr_0.1_AlO_3−δ_	800	9.0×10^−3^	0.93	[85]
La_0.8_Sr_0.2_AlO_3−δ_	800	6.2 × 10^−3^	1.06	[75]
La_0.8_Sr_0.2_AlO_3−δ_	900	1.5 × 10^−2^	1.06	[75]
La_0.8_Sr_0.2_AlO_3−δ_	900	1.1 × 10^−2^	1.16	[80]
La_0.8_Sr_0.2_AlO_3−δ_	810	4.3 × 10^−3^	1.06	[84]
La_0.7_Pr_0.2_Sr_0.1_AlO_3−δ_	800	2.3 × 10^−2^	0.84	[85]
LaAl_0.95_Zn_0.05_O_3−δ_	700	8.5 × 10^−4^	1.05	[62]
LaAl_0.95_Zn_0.05_O_3−δ_	900	1.1 × 10^−3^	1.05	[62]
LaAl_0.9_Mg_0.1_O_3−δ_	900	9.6 × 10^−3^	1.05	[80]
LaAl_0.5_Mn_0.5_O_3−δ_	800	4.7(2)	0.22	[75]
LaAl_0.5_Mn_0.5_O_3−δ_	900	5.8(2)	0.22	[75]
La_0.9_Sr_0.1_Al_0.9_Mg_0.1_O_3−δ_	700	2.6 × 10^−3^	1.56	[71]
La_0.9_Sr_0.1_Al_0.9_Mg_0.1_O_3−δ_	700	5.3 × 10^−4^	1.38	[88]
La_0.9_Sr_0.1_Al_0.9_Mg_0.1_O_3−δ_	900	2.0 × 10^−2^	0.90	[82]
La_0.8_Sr_0.2_Al_0.95_Mg_0.05_O_3−δ_	900	1.3 × 10^−2^	1.15	[80]
La_0.89_Sr_0.1_Ba_0.01_Al_0.9_Mg_0.1_O_3−δ_	700	2.6 × 10^−3^	1.48	[71]
La_0.89_Sr_0.1_Ba_0.01_Al_0.9_Mg_0.1_O_3−δ_ tape	700	6.0 × 10^−4^	0.60	[86]
La_0.89_Sr_0.1_Ba_0.01_Al_0.9_Mg_0.1_O_3−δ_ pellet	700	4.6 × 10^−2^	0.75	[86]
La_0.87_Sr_0.1_Ba_0.03_Al_0.9_Mg_0.1_O_3−δ_	700	1.7 × 10^−3^	1.38	[71]
La_0.8_Sr_0.2_Al_0.5_Mn_0.5_O_3−δ_	800	8.6(3)	0.15	[75]
La_0.8_Sr_0.2_Al_0.5_Mn_0.5_O_3−δ_	900	9.8(2)	0.15	[75]
La_0.8_Sr_0.2_Al_0.7_Mn_0.3_O_3−δ_	810	0.75	0.29	[84]
La_0.8_Sr_0.2_Al_0.5_Mn_0.5_O_3−δ_	810	10	0.17	[84]
(La_0.8_Sr_0.2_)_0.94_Al_0.5_Mn_0.5_O_3−δ_	810	12	0.14	[84]
La_0.9_Ba_0.1_Al_0.9_Y_0.1_O_3−δ_	800	1.8 × 10^−2^	0.82	[53]
La_0.9_Ba_0.1_Al_0.9_Y_0.1_O_3−δ_	900	3.1 × 10^−2^	0.82	[53]
La_0.87_Sr_0.1_Sm_0.03_Al_0.9_Mg_0.1_O_3−δ_	700	1.2 × 10^−3^	1.09	[88]
La_0.85_Sr_0.1_Sm_0.05_Al_0.9_Mg_0.1_O_3−δ_	700	1.1 × 10^−3^	1.10	[88]

**Table 2:** Total conductivities of LaGaO_3_-based materials depending on their compositions, preparation methods and temperatures. Figure A2 provides a visualization of these data.

Sample	Samples Obtaining Method; Annealing Temperature (°C)	*T* (°C)	*σ* (S cm^−1^)	Ref.
LaGaO_3_	Solid-state route; 1500	950	0.02	[51]
La_0.9_Sr_0.1_Ga_0.9_Mg_0.1_O_3−δ_	Solid-state route; 1500	950	0.20	[51]
La_0.9_Sr_0.1_Ga_0.85_Mg_0.15_O_3−δ_	Solid-state route; 1500	950	0.27	[51]
La_0.9_Sr_0.1_Ga_0.8_Mg_0.2_O_3−δ_	Solid-state route; 1500	950	0.29	[51]
La_0.9_Sr_0.1_Ga_0.7_Mg_0.3_O_3−δ_	Solid-state route; 1500	950	0.28	[51]
La_0.9_Sr_0.1_Ga_0.6_Mg_0.4_O_3−δ_	Solid-state route; 1500	950	0.10	[51]
La_0.9_Sr_0.1_Ga_0.8_Mg_0.2_O_3−δ_	Glycine-combustion method; 1400	1000	0.26	[51]
La_0.85_Sr_0.15_Ga_0.8_Mg_0.2_O_3−δ_	Glycine-combustion method; 1400	1000	0.36	[51]
La_0.8_Sr_0.2_Ga_0.85_Mg_0.15_O_3−δ_	Glycine-combustion method; 1400	1000	0.31	[51]
La_0.8_Sr_0.2_Ga_0.8_Mg_0.2_O_3−δ_	Glycine-combustion method; 1400	1000	0.40	[51]
La_0.9_Sr_0.1_Ga_0.9_Mg_0.1_O_3−δ_	Solid-state route; 1470	800	0.116	[95]
La_0.9_Sr_0.1_Ga_0.85_Mg_0.15_O_3−δ_	Solid-state route; 1470	800	0.127	[95]
La_0.9_Sr_0.1_Ga_0.8_Mg_0.2_O_3−δ_	Solid-state route; 1470	800	0.132	[95]
La_0.9_Sr_0.1_Ga_0.7_Mg_0.3_O_3−δ_	Solid-state route; 1470	800	0.096	[95]
La_0.85_Sr_0.15_Ga_0.8_Mg_0.2_O_3−δ_	Solid-state route; 1470	800	0.150	[95]
La_0.8_Sr_0.2_Ga_0.85_Mg_0.15_O_3−δ_	Solid-state route; 1470	800	0.149	[95]
La_0.8_Sr_0.2_Ga_0.83_Mg_0.17_O_3−δ_	Solid-state route; 1470	800	0.17	[95]
La_0.8_Sr_0.2_Ga_0.8_Mg_0.2_O_3−δ_	Solid-state route; 1470	800	0.14	[95]
La_0.7_Sr_0.3_Ga_0.8_Mg_0.2_O_3−δ_	Solid-state route; 1470	800	0.109	[95]
La_0.9_Sr_0.1_Ga_0.8_Mg_0.2_O_3−δ_	Self-propagating high-temperature synthesis; 1500	800	0.11	[110]
La_0.9_Sr_0.1_Ga_0.8_Mg_0.2_O_3−δ_	Carbonate co-precipitation; 1400	800	0.045	[104]
La_0.9_Sr_0.1_Ga_0.9_Mg_0.1_O_3−δ_	Solid-state route; 1450	800	0.071	[114]
La_0.9_Sr_0.1_Ga_0.8_Mg_0.2_O_3−δ_	Solid-state route; 1450	800	0.1095	[114]
La_0.9_Sr_0.1_Ga_0.8_Mg_0.2_O_3−δ_	Glycine-combustion method; 1500	800	0.092	[122]
La_0.9_Sr_0.1_Ga_0.8_Mg_0.2_O_3−δ_	Glycine-combustion method; 1400	800	0.0395	[123]
La_0.85_Sr_0.15_Ga_0.85_Mg_0.15_O_3−δ_	Acrylamide polymerization technique; 1432	800	0.093	[124]
La_0.85_Sr_0.15_Ga_0.8_Mg_0.2_O_3−δ_	Mechanochemical route; 1380	600	0.016	[97]
La_0.85_Sr_0.15_Ga_0.8_Mg_0.2_O_3−δ_	Glycine-combustion method; 1300	800	0.053	[125]
La_0.85_Sr_0.15_Ga_0.8_Mg_0.2_O_3−δ_	EDTA-combustion method; 1300	800	0.06	[125]
La_0.85_Sr_0.15_Ga_0.8_Mg_0.2_O_3−δ_	Glycine-combustion method; 1400	800	0.096	[105]
La_0.85_Sr_0.15_Ga_0.8_Mg_0.2_O_3−δ_	Pechini method; 1400	800	0.135	[126]
La_0.8_Sr_0.2_Ga_0.8_Mg_0.2_O_3−δ_	Carbonate co-precipitation; 1300	600	0.014	[103]
La_0.8_Sr_0.2_Ga_0.8_Mg_0.2_O_3−δ_	Glycine-combustion method; 1300	700	0.022	[109]
La_0.8_Sr_0.2_Ga_0.8_Mg_0.2_O_3−δ_	Glycine-combustion method; 1400	700	0.085	[109]
La_0.8_Sr_0.2_Ga_0.8_Mg_0.2_O_3−δ_	Spray pyrolysis; 1400	500	0.0029	[112]
La_0.8_Sr_0.2_Ga_0.8_Mg_0.2_O_3−δ_	Solid-state route; 1450	800	0.126	[127]
La_0.8_Sr_0.2_Ga_0.8_Mg_0.2_O_3−δ_	Solid-state route; 1400	800	0.035	[127]
La_0.8_Sr_0.2_Ga_0.8_Mg_0.2_O_3−δ_	Hydrothermal urea hydrolysis precipitation; 1400	800	0.056	[127]
La_0.8_Sr_0.2_Ga_0.8_Mg_0.2_O_3−δ_	Carbonate co-precipitation; 1400	800	0.137	[128]
La_0.8_Sr_0.2_Ga_0.8_Mg_0.2_O_3−δ_	Solid-state route; 1250	727	0.019	[129]
La_0.8_Sr_0.2_Ga_0.8_Mg_0.2_O_3−δ_	Sol-gel technique; 1300	450	2.9 × 10^−4^	[130]
La_0.8_Sr_0.2_Ga_0.8_Mg_0.2_O_3−δ_	Solid-state route; 1400	800	0.132	[131]
La_0.8_Sr_0.2_Ga_0.8_Mg_0.2_O_3−δ_	Thin film deposited by vacuum cold spray; 200	750	0.043	[132]
La_0.8_Sr_0.2_Ga_0.8_Mg_0.2_O_3−δ_	Step-wise current-limiting flash sintering process; 690	850	0.072	[133]

**Table 3:** The performances of SOFCs with La_1__−*x*_Sr*_x_*Ga_1−*y*_Mg*_y_*O_3−δ_ electrolytes. Figure A3 provides a visualization of these data.

Anode	Buffer Layer/ Electrolyte (Thickness, μm)/ Buffer Layer	Cathode	*T* (°C)	Power Density (mW cm^−2^)	Ref.
Ni-Ce_0.8_Sm_0.2_O_2−δ_	La_0.8_Sr_0.2_Ga_0.83_Mg_0.17_O_3−δ_ (265)	La_0.6_Sr_0.4_O_3−δ_	800	290	[52]
Ni-La_0.8_Sr_0.2_Ga_0.83_Mg_0.17_O_2.815_	La_0.8_Sr_0.2_Ga_0.83_Mg_0.17_O_3−δ_ (395)	La_0.6_Sr_0.4_O_3−δ_	800	363	[52]
Ni-Ce_0.8_Sm_0.2_O_2−δ_	Ce_0.8_Sm_0.2_O_2−δ_/La_0.9_Sr_0.1_Ga_0.8_Mg_0.2_O_3−δ_ (300)	La_0.4_Sr_0.6_Co_0.9_Sb_0.1_O_3−δ_- Ce_0.8_Sm_0.2_O_2−δ_	700	432	[144]
Ni-Ce_0.8_Sm_0.2_O_2−δ_	Ce_0.8_Sm_0.2_O_2−δ_/La_0.9_Sr_0.1_Ga_0.8_Mg_0.2_O_3−δ_ (100)	SrCo_0.8_Fe_0.1_Nb_0.1_O_3−δ_	800	756	[145]
Ni-Ce_0.8_Sm_0.2_O_2−δ_	Ce_0.8_Sm_0.2_O_2−δ_/La_0.9_Sr_0.1_Ga_0.8_Mg_0.2_O_3−δ_ (100)	SrCo_0.8_Fe_0.1_Nb_0.1_O_3−δ_– Ce_0.9_Gd_0.1_O_2−δ_	800	829	[145]
Ni-Ce_0.8_Sm_0.2_O_2−δ_	La_0.9_Sr_0.1_Ga_0.8_Mg_0.2_O_3−δ_ (300)	BaCo_0.7_Fe_0.2_Ta_0.1_O_3−δ_	800	460	[146]
Ni-Ce_0.8_Sm_0.2_O_2−δ_	Ce_0.8_Sm_0.2_O_2−δ_/La_0.9_Sr_0.1_Ga_0.8_Mg_0.2_O_3−δ_ (300)	Pr_2_Ni_0.85_Cu_0.1_Al_0.05_O_4+δ_	700	392	[149]
Ni-Ce_0.8_Sm_0.2_O_2−δ_	La_0.8_Sr_0.2_Ga_0.83_Mg_0.17_O_3−δ_ (500)	La_0.6_Sr_0.4_O_3−δ_	800	270	[155,173]
Ni-Ce_0.8_Sm_0.2_O_2−δ_	Ce_0.8_Sm_0.2_O_2−δ_/La_0.8_Sr_0.2_Ga_0.83_Mg_0.17_O_3−δ_ (500)	La_0.6_Sr_0.4_O_3−δ_	800	550	[155,173]
Ni-Ce_0.8_Sm_0.2_O_2−δ_	La_0.87_Sr_0.13_Ga_0.85_Mg_0.15_O_3−δ_ (3.8)	La_0.87_Sr_0.13_Ga_0.85_Mg_0.15_O_3−δ_- La_0.6_Sr_0.4_Fe_0.8_Co_0.2_O_3−δ_	750	1420	[174]
Ni-Ce_0.8_Y_0.2_O_2−δ_	La_0.9_Sr_0.1_Ga_0.8_Mg_0.2_O_3−δ_ (45)	La_0.6_Sr_0.4_O_3−δ_	700	500	[175]
Ni-Ce_0.6_La_0.4_O_2−δ_	La_0.8_Sr_0.2_Ga_0.8_Mg_0.2_O_3−δ_ (30)	La_0.8_Sr_0.2_Fe_0.8_Co_0.2_O_3−δ_	700	780	[176]
Ni-Ce_0.6_La_0.4_O_2−δ_	Ce_0.6_La_0.4_O_2−δ_/La_0.8_Sr_0.2_Ga_0.83_Mg_0.17_O_3−δ_ (500)	SrCo_0.8_Fe_0.2_O_3−δ_	800	900	[177]
Ni-Ce_0.9_Gd_0.1_O_2−δ_	Ce_0.55_La_0.45_O_2−δ_/La_0.9_Sr_0.1_Ga_0.8_Mg_0.2_O_3−δ_ (75)	La_0.9_Sr_0.1_O_3−δ_- Ce_0.55_La_0.45_O_2−δ_	800	1100	[178]
Ni-Ce_0.9_Gd_0.1_O_2−δ_	Ce_0.55_La_0.45_O_2−δ_/La_0.9_Sr_0.1_Ga_0.8_Mg_0.2_O_3−δ_ (50)	La_0.6_Sr_0.4_O_3−δ_	800	1565	[179]
Ni-Ce_0.9_Gd_0.1_O_2−δ_	Ce_0.55_La_0.45_O_2−δ_/La_0.9_Sr_0.1_Ga_0.8_Mg_0.2_O_3−δ_ (50)/Ce_0.55_La_0.45_O_2−δ_	La_0.6_Sr_0.4_O_3−δ_	800	871	[179]
Ni-Ce_0.8_Gd_0.2_O_2−δ_	Ce_0.8_Gd_0.2_O_2−δ_/La_0.9_Sr_0.1_Ga_0.8_Mg_0.2_O_3−δ_ (75)	Ba_0.5_Sr_0.5_Co_0.8_Fe_0.2_O_3−δ_	700	760	[180]
Ni-Fe	Ce_0.8_Sm_0.2_O_2−δ_/La_0.9_Sr_0.1_Ga_0.8_Mg_0.2_O_3−δ_ (6)	Sm_0.5_Sr_0.5_O_3−δ_	700	1790	[181]
Ni-Ce_0.6_La_0.4_O_2−δ_	Ce_0.6_La_0.4_O_2−δ_/La_0.9_Sr_0.1_Ga_0.8_Mg_0.2_O_3−δ_ (9)/Ce_0.6_La_0.4_O_1.8_	La_0.9_Sr_0.1_Ga_0.8_Mg_0.2_O_3−δ_- La_0.6_Sr_0.4_Fe_0.8_Co_0.2_O_3−δ_	700	910	[182]
Ni-Ce_0.8_Sm_0.2_O_2−δ_	Ce_0.6_La_0.4_O_2−δ_/La_0.9_Sr_0.1_Ga_0.8_Mg_0.2_O_3−δ_ (11)/Ce_0.6_La_0.4_O_1.8_	La_0.9_Sr_0.1_Ga_0.8_Mg_0.2_O_3−δ_- La_0.6_Sr_0.4_Fe_0.8_Co_0.2_O_3−δ_	800	1230	[183]
Ni-Ce_0.8_Gd_0.2_O_2−δ_	Ce_0.8_Gd_0.2_O_2−δ_/(La_0.9_Sr_0.1_)_0.97_Ga_0.9_Mg_0.1_O_3−δ_ (120)	La_0.6_Sr_0.4_Fe_0.8_Co_0.2_O_3−δ_	800	540	[170]
Ni-Ce_0.8_Sm_0.2_O_2−δ_	La_0.9_Sr_0.1_Ga_0.8_Mg_0.2_O_3−δ_ (3.4)	La_0.9_Sr_0.1_Ga_0.8_Mg_0.2_O_3−δ_- La_0.6_Sr_0.4_Fe_0.8_Co_0.2_O_3−δ_	750	736	[184]
Ni-Ce_0.8_Gd_0.2_O_2−δ_	La_0.8_Sr_0.2_Ga_0.8_Mg_0.2_O_3−δ_ (50)	La_0.6_Sr_0.4_Fe_0.8_Co_0.2_O_3−δ_	700	831	[185]
Ni-Fe	Ce_0.6_La_0.4_O_2−δ_/La_0.9_Sr_0.1_Ga_0.8_Mg_0.2_O_3−δ_ (200)	Sm_0.5_Sr_0.5_O_3−δ_	800	1350	[171]
Pd-Sr_2_TiMoO_6−δ_	Ce_0.8_Sm_0.2_O_2−δ_/La_0.9_Sr_0.1_Ga_0.8_Mg_0.2_O_3−δ_ (300)	NdBaCo_0.67_Fe_0.67_Cu_0.67_O_5+δ_	850	1009	[160]
Sr_2_NiMoO_6−δ_	La_0.88_Sr_0.12_Ga_0.82_Mg_0.18_O_3−δ_ (700)/Ce_0.8_Sm_0.2_O_2−δ_	La_0.7_Sr_0.3_Fe_0.9_Co_0.1_O_3−δ_	800	61	[163]
Sr_2_NiMoO_6−δ_	La_0.9_Sr_0.1_Ga_0.8_Mg_0.2_O_3−δ_ (300)	Ba_0.5_Sr_0.5_Co_0.8_Fe_0.2_O_3−δ_	800	595	[186]
Sr_2_MgMoO_6−δ_	Ce_0.8_Sm_0.2_O_2−δ_/La_0.8_Sr_0.2_Ga_0.8_Mg_0.2_O_3−δ_ (700)	SmBaCo_2_O_5+δ_	800	39	[167]
Sr_2_MgMoO_6−δ_	Ce_0.8_Gd_0.2_O_2−δ_/La_0.8_Sr_0.2_Ga_0.8_Mg_0.2_O_3−δ_ (600)	La_0.6_Sr_0.4_Fe_0.8_Co_0.2_O_3−δ_	800	330	[168]
Sr_2_Ni_0.75_Mg_0.25_MoO_6−δ_	La_0.88_Sr_0.12_Ga_0.82_Mg_0.18_O_3−δ_ (700)/Ce_0.8_Sm_0.2_O_2−δ_	La_0.7_Sr_0.3_Fe_0.9_Co_0.1_O_3−δ_	800	429	[163]
Sr_2_Ni_0.75_Mg_0.25_MoO_6−δ_	La_0.88_Sr_0.12_Ga_0.82_Mg_0.18_O_3−δ_ (500)/Ce_0.8_Sm_0.2_O_2−δ_	La_2_NiO_4+δ_	800	276	[187]
Sr_2_Ni_0.75_Mg_0.25_MoO_6−δ_	La_0.88_Sr_0.12_Ga_0.82_Mg_0.18_O_3−δ_ (500)/Ce_0.8_Sm_0.2_O_2−δ_	La_1.5_Ca_0.5_Ni_0.67_Fe_0.33_O_4+δ_	800	273	[187]
Sr_2_Ni_0.7_Mg_0.3_MoO_6−δ_	Ce_0.8_Sm_0.2_O_2−δ_/La_0.8_Sr_0.2_Ga_0.8_Mg_0.2_O_3−δ_ (700)	SmBaCo_2_O_5+δ_	800	160	[167]
Sr_2_Ni_0.3_Mg_0.7_MoO_6−δ_	Ce_0.8_Sm_0.2_O_2−δ_/La_0.8_Sr_0.2_Ga_0.8_Mg_0.2_O_3−δ_ (700)	SmBaCo_2_O_5+δ_	800	119	[167]
Ba_0.5_Sr_0.5_Mo_0.1_Fe_0.9_O_3−δ_	La_0.8_Sr_0.2_Ga_0.8_Mg_0.2_O_3−δ_ (150)	Ba_0.5_Sr_0.5_Mo_0.1_Fe_0.9_O_3−δ_	800	2280	[188]
SrFe_0.__75_Mo_0.__25_O_3−δ_	La_0.9_Sr_0.1_Ga_0.8_Mg_0.2_O_3−δ_ (30)	SrFe_0.__75_Mo_0.__25_O_3−δ_	800	703	[189]
PrBa(Fe_0.8_Sc_0.2_)_2_O_5+δ_	La_0.9_Sr_0.1_Ga_0.8_Mg_0.2_O_3−δ_ (275)	PrBa(Fe_0.8_Sc_0.2_)_2_O_5+δ_	800	713	[190]
Sr_2_Fe_1.5_Mo_0.5_O_6−δ_- La_0.9_Sr_0.1_Ga_0.8_Mg_0.2_O_2.85_	La_0.9_Sr_0.1_Ga_0.8_Mg_0.2_O_3−δ_ (10)	Sr_2_Fe_1.5_Mo_0.5_O_6−δ_- La_0.9_Sr_0.1_Ga_0.8_Mg_0.2_O_3−δ_	700	880	[191]
Pr_0.6_Sr_0.4_Fe_0.8_Ni_0.2_O_3−δ_	Ce_0.8_Gd_0.2_O_2−δ_/La_0.9_Sr_0.1_Ga_0.8_Mg_0.2_O_3−δ_ (320)/Ce_0.8_Gd_0.2_O_1.9_	Pr_0.6_Sr_0.4_Fe_0.8_Ni_0.2_O_3−δ_	800	500	[192]
PrBaMn_1.5_Fe_0.5_O_5+δ_	La_0.8_Sr_0.2_Ga_0.8_Mg_0.2_O_3−δ_ (520)	PrBaMn_1.5_Fe_0.5_O_5+δ_	800	540	[193]
La_0.5_Sr_0.5_Fe_0.9_Nb_0.1_O_3−δ_	La_0.82_Sr_0.18_Ga_0.83_Mg_0.17_O_3−δ_ (300)	La_0.5_Sr_0.5_Fe_0.9_Nb_0.1_O_3−δ_	750	630	[194]
La_0.54_Sr_0.36_Co_0.2_Fe_0.6_Nb_0.2_O_3−δ_	Ce_0.8_Sm_0.2_O_2−δ_/La_0.9_Sr_0.1_Ga_0.8_Mg_0.2_O_3−δ_ (200)/Ce_0.8_Sm_0.2_O_1.9_	La_0.54_Sr_0.36_Co_0.2_Fe_0.6_Nb_0.2_O_3−δ_	800	539	[195]
Sr_2_TiFe_0.9_Mo_0.1_O_6−δ_	Ce_0.8_Sm_0.2_O_2−δ_/La_0.9_Sr_0.1_Ga_0.8_Mg_0.2_O_3−δ_ (200)/Ce_0.8_Sm_0.2_O_1.9_	Sr_2_TiFe_0.9_Mo_0.1_O_6−δ_	800	444	[196]
Sr_2_Fe_1.4_Nb_0.1_Mo_0.5_O_6−δ_	La_0.8_Sr_0.2_Ga_0.83_Mg_0.17_O_3−δ_ (243)	Sr_2_Fe_1.4_Nb_0.1_Mo_0.5_O_6−δ_	800	531	[197]
Sr_0.95_Ti_0.3_Fe_0.63_Ni_0.07_O_3−δ_	Ce_0.6_La_0.4_O_2−δ_/La_0.8_Sr_0.2_Ga_0.83_Mg_0.17_O_3−δ_ (300)	La_0.6S_Sr_0.4_Co_0.2_Fe_0.8_O_3−δ_- Gd_0.1_Ce_0.9_O_2−δ_	800	1000	[172]

## Data Availability

Not applicable.
